# Shared Molecular Mechanisms of Hypertrophic Cardiomyopathy and Its Clinical Presentations: Automated Molecular Mechanisms Extraction Approach

**DOI:** 10.3390/life11080785

**Published:** 2021-08-03

**Authors:** Mila Glavaški, Lazar Velicki

**Affiliations:** 1 Faculty of Medicine, University of Novi Sad, Hajduk Veljkova 3, 21000 Novi Sad, Serbia; lazar.velicki@mf.uns.ac.rs; 2 Institute of Cardiovascular Diseases Vojvodina, Put Doktora Goldmana 4, 21204 Sremska Kamenica, Serbia

**Keywords:** hypertrophic cardiomyopathy, data mining, automated curation, molecular mechanisms, atrial fibrillation, sudden cardiac death, heart failure, left ventricular outflow tract obstruction, cardiac fibrosis, myocardial ischemia

## Abstract

Hypertrophic cardiomyopathy (HCM) is the most common inherited cardiovascular disease with a prevalence of 1 in 500 people and varying clinical presentations. Although there is much research on HCM, underlying molecular mechanisms are poorly understood, and research on the molecular mechanisms of its specific clinical presentations is scarce. Our aim was to explore the molecular mechanisms shared by HCM and its clinical presentations through the automated extraction of molecular mechanisms. Molecular mechanisms were congregated by a query of the INDRA database, which aggregates knowledge from pathway databases and combines it with molecular mechanisms extracted from abstracts and open-access full articles by multiple machine-reading systems. The molecular mechanisms were extracted from 230,072 articles on HCM and 19 HCM clinical presentations, and their intersections were found. Shared molecular mechanisms of HCM and its clinical presentations were represented as networks; the most important elements in the intersections’ networks were found, centrality scores for each element of each network calculated, networks with reduced level of noise generated, and cooperatively working elements detected in each intersection network. The identified shared molecular mechanisms represent possible mechanisms underlying different HCM clinical presentations. Applied methodology produced results consistent with the information in the scientific literature.

## 1. Introduction

Hypertrophic cardiomyopathy (HCM) is the most common inherited cardiovascular disease [1,2,3], with an estimated prevalence of 1 in 500 people worldwide [1,3,4,5] and recent investigations suggesting an even greater prevalence [5,6]. It is characterized by increased left ventricular wall thickness that cannot be explained by abnormal loading conditions (e.g., hypertension) [1,2,7].

Mutations in genes that encode sarcomeric proteins are the primary molecular cause of HCM [3,8,9]. However, the genetic basis for HCM has proven to be more complex than originally postulated: 40–60% of HCM patients show mutations in one or more of the genes known to be associated with the disease, whereas for others, the cause remains unknown [10,11,12].

The clinical presentation of HCM varies widely [1,3,7,8]: some patients are asymptomatic [1,7,13], while others manifest symptomatic left ventricular outflow tract obstruction (LVOTO) [7,8], atrial fibrillation (AF) [3,8], sudden cardiac death (SCD) [3,7,13,14], or heart failure (HF) [1,3,10,11]. Pathophysiologic features of HCM include cardiomyocyte hypertrophy [15,16], cardiomyocyte disarray [16,17], myocardial remodeling [18,19], fibrosis [3,20,21], myocardial hypercontractility [22,23], impaired myocardial relaxation [20,24], myocardial stiffness [17,20], diastolic dysfunction [13,14,17], coronary microvascular dysfunction [25,26], and myocardial ischemia [25,27], but the underlying molecular mechanisms are poorly understood. Molecular determinants of the disease presentations are also still not known. Phenotypic expression of HCM may vary even within the same family [1]. Despite active research, the consistent genotype-phenotype associations are still not known. All these stress the importance of finding additional mechanisms and factors that direct the course and presentations of HCM, and propose all the molecular mechanisms standing between genetic basis and clinical presentations as crucial.

INDRA database [28] aggregates knowledge from pathway databases and combines it with information on molecular mechanisms extracted from abstracts and open-access full articles by multiple machine-reading systems. PubMed is one of the most important platforms for medical journal literature. To be indexed in PubMed, journals must meet certain review or selection criteria [29].

Our aim was to explore the shared molecular mechanisms of HCM and its clinical presentations through the automated extraction of molecular mechanisms.

## 2. Materials and Methods

### 2.1. Molecular Mechanisms Extraction

Molecular mechanisms were congregated using the INDRA database [28]. Molecular mechanisms from all PubMed articles published starting from 1 January 2010 were separately extracted in the form of INDRA statements [30] for HCM, cardiomyocyte hypertrophy, myofibrillar disarray, cardiomyocyte disarray, myocardial remodeling, cardiac remodeling, myocardial fibrosis, LVOTO, myocardial hypercontractility, impaired myocardial relaxation, impaired cardiac relaxation, myocardial stiffness, diastolic dysfunction, AF, SCD, coronary microvascular dysfunction, myocardial ischemia, HF, MACE, and rehospitalization. INDRA statements were found in the INDRA database by PubMed Identifiers (PMIDs), using REST Client API. PMIDs were collected through the INDRA PubMed client [30] (which searches for articles on PubMed) using the following search terms: hypertrophic cardiomyopathy, cardiomyocyte hypertrophy, myofibrillar disarray, cardiomyocyte disarray, myocardial remodeling, cardiac remodeling, myocardial fibrosis, left ventricular outflow tract obstruction, myocardial hypercontractility, impaired myocardial relaxation, impaired cardiac relaxation, myocardial stiffness, diastolic dysfunction, atrial fibrillation, sudden cardiac death, coronary microvascular dysfunction, myocardial ischemia, heart failure, major adverse cardiovascular events, and rehospitalization (use_text_word = True, major_topic = True).

Subsequently, intersections of the sets consisting of INDRA statements for HCM and its clinical presentations were found.

### 2.2. Networks Generation

Each of the intersections (consisting of sets of INDRA statements) was transcribed to a network table, imported to Cytoscape version 3.8.2 [31] for further analysis, and uploaded to NDEx v 2.5.0 [32,33,34].

### 2.3. Network Analysis

The most important nodes in intersections’ networks were found using Cytoscape application Wk shell decomposition version 1.1.0 [35]. Rank and k-shell were calculated for every node of each network. The reliability of interactions was determined using Cytoscape PE-measure application version 1.0 [36]. Models with a reduced level of noise were generated and uploaded to NDEx. The nodes’ centrality scores were determined using Cytoscape CytoHubba app version 0.1. [37]. Top elements for each centrality measure of each network were uploaded to NDEx. Cooperatively working elements (functional modules) were found using NCMine Cytoscape plugin version 1.3.0 [38]. All networks were analyzed as directed (with applied cliqueness threshold = 0.6, merge threshold = 0.6, dcliqueness threshold = 0.2, and cluster size threshold = 3).

## 3. Results

Molecular mechanisms in the form of 182,167 INDRA statements (representations of molecular mechanisms consisting molecular subject, object, and their interaction) were extracted from 230,072 articles on HCM and 19 HCM clinical presentations (Table 1).

### 3.1. Network Analysis

#### 3.1.1. Networks

Shared molecular mechanisms of HCM and its clinical presentations are represented as networks (Table 2). The networks differ notably in terms of the number of elements they contain.

The intersection of molecular interactions representing HCM and impaired cardiac relaxation contains only phosphorylation of SMAD Family Member 2 (SMAD2) and could not be displayed as a network. The intersection of HCM and myocardial hypercontractility contains no molecular interactions.

#### 3.1.2. The Most Important Nodes

The most important nodes for all networks are found (Supplementary Table S1). All networks were presented as packed concentric rings sorted by the most important nodes (Supplementary Figure S1).

#### 3.1.3. Nodes’ Centrality Scores

Centrality scores for each node of each network were calculated, and the top elements for each centrality measure of each network were visualized (Table 3).

#### 3.1.4. Reliability of Interactions

Networks with a reduced level of noise were generated (Table 4).

#### 3.1.5. Cooperatively Working Elements

In each intersection network, cooperatively working elements (functional modules) were detected (Supplementary Table S2).

### 3.2. Shared Molecular Elements and Pathways

#### 3.2.1. Hypertrophic Cardiomyopathy and Structural Changes

The most important shared elements for cardiomyocyte hypertrophy and HCM were as follows: calcium; Ca^2+^/calmodulin-dependent protein kinase II (CaMKII); *PLN* gene encoding phospholamban; protein kinase A (PKA), which is a master regulator of most cAMP-dependent processes; protein kinase B (PKB), also known as AKT, which regulates cellular survival and metabolism; AMP-activated protein kinase (AMPK), which is involved in cellular energy homeostasis as a “cellular energy sensor”; and sirtuin 1 encoded by *SIRT1* gene; nuclear factor of activated T-cells (NFAT), which is important for immune response and is involved in the development of the cardiac system; *EDN1* gene encoding endothelin 1 (ET-1), which is a potent vasoconstrictor; *AGT* gene, which encodes angiotensinogen; collagen; multifunctional cytokine transforming growth factor- β (TGF-β); signal transduction protein extracellular signal-regulated kinase (ERK); cell population proliferation; and apoptosis.

The most important shared elements for myofibrillar disarray and HCM are actin, myosin complex, *MYL12A* gene, *MYBPC3* gene, ATP, mitogen-activated protein kinase 7 (MAPK7) encoded by the *MAPK7* gene (MAP kinases are involved in many cellular processes), RAF proto-oncogene serine/threonine-protein kinase (RAF1)—a part of the ERK1/2 pathway as a MAP kinase encoded by *RAF1* gene, ERK, and *EDN1* gene encoding endothelin 1. The effects of immunosuppressant and calcineurin inhibitor cyclosporin A as well as MAP kinase cascade inhibitor PD98059 are also indicated.

In their pathophysiology, cardiomyocyte disarray and HCM share the mechanisms of contractile machinery components (actin, myosin complex, and enzyme ATPase); apoptosis-inhibiting mechanisms (B-cell lymphoma 2 gene, *BCL2*); the protein tyrosine phosphatase non-receptor type 11 (PTPN11), which inhibits the growth regulator—the mechanistic target of rapamycin, mTOR; and Src homology 2 domain-containing phosphatase 2 (Shp2), which is involved in cell growth and survival. The importance of the myosin heavy chain 7 gene, *MYH7*, is shown.

The common molecular elements of myocardial remodeling and HCM were as follows: calcium, CaMKII, *AGT* gene, which encodes angiotensinogen, angiotensin II, collagen, TGF-β, tumor necrosis factor (TNF), inflammatory response, cell population proliferation, and apoptosis.

Cardiac remodeling and HCM in their pathophysiology share calcium, AMPK (a “cellular energy sensor”), *AGT* gene encoding angiotensinogen, AKT (regulates cellular survival and metabolism), TGF-β (multifunctional cytokine), *SIRT1* gene encoding sirtuin 1 (SIRT1), collagen, actin, reactive oxygen species, cell population proliferation, and apoptosis.

By the most important nodes and ranked by centrality scores, the most important shared elements for myocardial fibrosis and HCM were calcium, TGF-β, collagen, *AGT* encoding angiotensinogen, angiotensin II, AMPK, cell population proliferation, inflammatory response, and apoptosis.

#### 3.2.2. Hypertrophic Cardiomyopathy and Left Ventricular Outflow Tract Obstruction

LVOTO and HCM share calcium, TGF-β, *POSTN* gene encoding periostin (extracellular matrix protein with multiple functions), collagen, *PIMREG* gene and PIMREG protein (involved in metaphase-to-anaphase transition during mitosis), *SNCG* gene encoding gamma-synuclein (a member of the synuclein family of proteins, which were believed to be involved in the pathogenesis of neurodegenerative diseases and certain tumors), verapamil (calcium channel blocker), dobutamine (β1-agonist), mavacamten (MYK-461, inhibitor of cardiac myosin ATPase), systolic anterior motion, and death.

#### 3.2.3. Hypertrophic Cardiomyopathy and Contractile Dysfunction

Impaired myocardial relaxation and HCM in their pathophysiology share nitric oxide (NO) and constitutive nitric oxide synthase (also known as nitic oxide synthase 3 (NOS3) or endothelial NOS) encoded by the *NOS3* gene as well as N, N-dimethylarginine, a direct endogenous inhibitor of NO synthases; interaction of phospholamban and ATP2A2 intracellular calcium pump; and collagen induction by TGF-β and PKA.

Molecules shared by myocardial stiffness and HCM were the *TTN* gene encoding titin, *RBM20* gene encoding RNA-binding protein that acts as a regulator of mRNA splicing of a subset of genes involved in cardiac development (regulates splicing of *TTN*), actin, TGF-β, *PRKCA* gene encoding protein kinase C-alpha (PKC-α), which was involved in diverse cellular signaling pathways, cyclic GMP-dependent protein kinase (PRKG), which involved in muscle relaxation, *RING1* gene encoding ring finger protein 1 (RING1), and *TRIM63* gene encoding tripartite motif-containing protein 63, which regulates the proteasomal degradation of muscle proteins.

Diastolic dysfunction and HCM share calcium, sodium, actin, troponin I, *TTN* gene encoding titin, *PLN* gene encoding phospholamban, cardiac myosin binding protein-C (cMyBP-C), CaMKII, TGF-β, PKA, AMPK, and apoptosis.

#### 3.2.4. Hypertrophic Cardiomyopathy and Arrhythmia

Based on the most important nodes and centrality score ranks, the following elements were the most important shared elements for AF and HCM: sodium and calcium; CaMKII, which is important for calcium homeostasis in cardiomyocytes; *PLN* gene encoding phospholamban that inhibits the activity of ATPase sarcoplasmic/endoplasmic reticulum Ca^2+^ transporting 2 (*ATP2A2*—encodes one of the intracellular pumps that return calcium from the cytosol to the sarcoplasmic reticulum); *RYR2* gene encoding ryanodine receptor 2 (RyR2) (major mediator in calcium-induced calcium release from sarcoplasmic reticulum); *AGT* gene which encodes angiotensinogen, a precursor of angiotensin; junctophilin 2 (*JPH2*) gene which encodes a component of junctional complexes (it also plays a key role in calcium-induced calcium release); T-cell leukemia homeobox protein 2 (*TLX2*) gene; *PSMD4* gene which encodes component of the 26S proteasome, with its main role being the removal of misfolded or damaged proteins as well as proteins whose functions are no longer required. Both diseases share inflammatory response, apoptotic process, and death in their pathophysiology.

SCD and HCM share the following elements: sodium, calcium, *RYR2* gene encoding RyR2, CaMKII, actin, *MYL12A* gene, myosin complex, *TNNT1* gene encoding troponin T, troponin C, *TNNI3* gene encoding troponin I, ATP, PKA, *GJA1* gene encoding gap junction protein alpha 1 (connexin-43), *PLN* gene encoding phospholamban, *GSTK1* gene encoding glutathione S-transferase kappa 1, which belongs to a superfamily of enzymes for cellular detoxification, and death. The effect of the non-selective β adrenoceptor agonist isoprenaline is also indicated.

#### 3.2.5. Hypertrophic Cardiomyopathy and Ischemia

The important common molecular mechanisms of coronary microvascular dysfunction and HCM are the serin/threonine-specific protein kinase Akt (also known as protein kinase B or PKB, this plays an important role in glucose metabolism, cell proliferation, and apoptosis) and its activating phosphorylation site S473, structural sarcomeric protein titin, components of the phosphagen energy system (ATP decreased by creatine), glucose increased by insulin, calcium increased by sodium, and calcium increased by calcium. The effects of a few exogenous elements, such as antioxidant resveratrol (which activates the sirtuin 1 gene, *SIRT1*, regulator of whole-body lipid homeostasis), antimineralocorticoid spironolactone (inhibiting expression of mineralocorticoid receptor, encoded by nuclear receptor subfamily 3 group C member 2 gene, *NR3C2*), and insecticide pyraclofos (increasing calcium) are also indicated.

Myocardial ischemia and HCM share the following elements: calcium, ATP, AMPK, PKA, AKT cross-talking with ERK, glucose, *INS*, *SIRT1* gene encoding sirtuin 1, NF-κB, TNF, reactive oxygen species, inflammatory response, and apoptosis.

#### 3.2.6. Hypertrophic Cardiomyopathy and Endpoints

In their pathogenesis, HF and HCM share calcium, sodium, RYR2, components of contractile machinery and related genes actin, myosin complex, myosin light chain 12A (*MYL12A*) gene, troponin C, troponin T, *TNNI3* gene encoding troponin I, tropomyosin, myosin binding protein C3 (*MYBPC3*) gene, cMyBP-C, glucose, *INS* gene encoding insulin, reactive oxygen species, ATP, 3’,5’-cyclic AMP (cAMP), ATPase, AMPK, sirtuin 1, TGF-β, collagen, *AGT* gene encoding angiotensinogen, *EDN1* gene encoding endothelin-1, ERK, *GATA4* encoding transcription factor GATA-4, PKA, PKB (AKT), protein kinase C (PKC), p38 MAP kinase, beta adrenergic receptor genes (ADRBs), mechanistic target of rapamycin (mTOR), NF-κB, calmodulin, CaMKII, CaMKII-delta, *PLN* gene encoding phospholamban, *TTN* gene encoding titin, *CLEC3B* gene encoding tetranectin, *E2F1*, *PSMD4*, and *SMARCA4* genes, NFAT, β adrenoceptor agonist isoprenaline, inflammatory response, autophagy, cell population proliferation, apoptosis, and death.

Major adverse cardiovascular events (MACE) and HCM in their pathophysiology share calcium, (R)-lipoic acid (the most active isomer of a versatile antioxidant, alpha-lipoic acid), *NR3C2* gene encoding mineralocorticoid receptor, and ATPase (a class of enzymes that catalyze the hydrolysis of ATP to ADP). Apart from this, interesting elements found in the intersection and ranked as top nodes for some centrality measures are insulin, *PLN* gene encoding phospholamban, *PSMD4* gene, and TGF-β.

Rehospitalization and HCM share the following aspects: insulin receptor, glucose decrease mediated by insulin; ryanodine receptor; *PSMD4* gene related to increased death; *NLRP3* gene encoding regulator of immunity and inflammation cryopyrin (also known as angiotensin/vasopressin receptor AII/AVP-like), promoting proinflammatory cytokine interleukin 1 beta (IL-1B).

#### 3.2.7. The Most Important Shared Elements and Pathways

**Figure 1:**
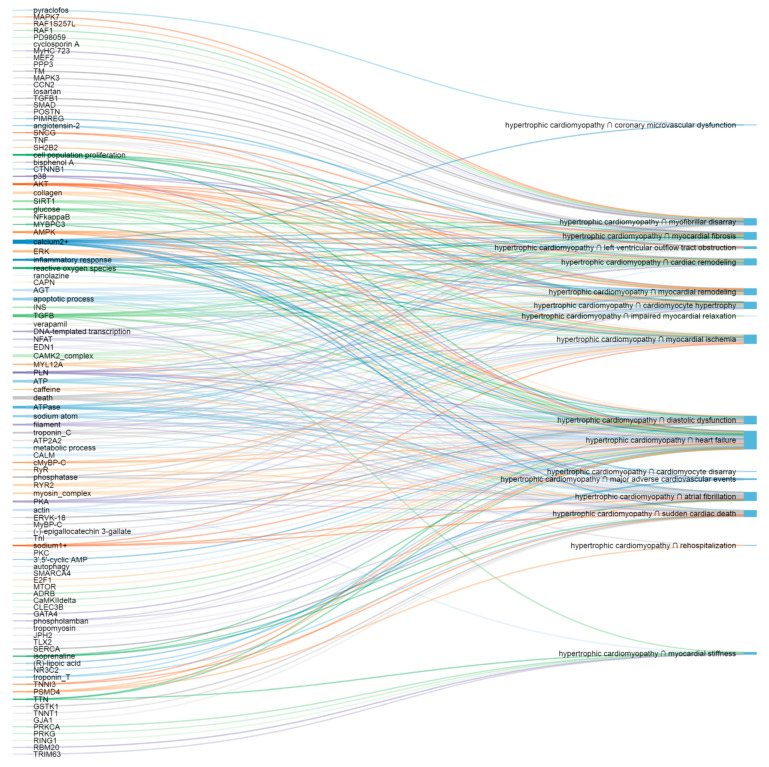
The most important putative molecular elements (**left**) and corresponding HCM presentations (**right**).

**Figure 2:**
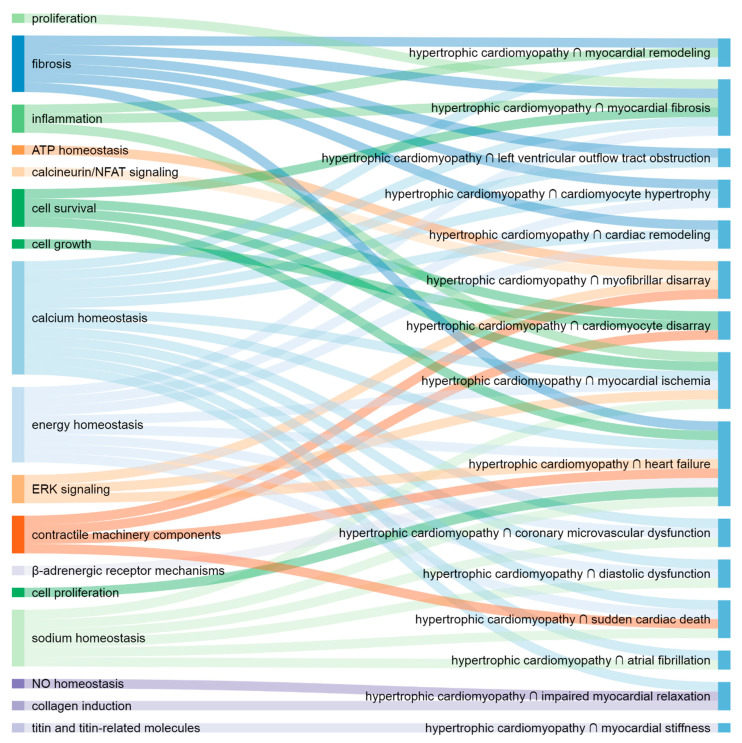
The most important putative pathways (**left**) and corresponding HCM presentations (**right**).

The most important putative molecular elements and pathways are illustrated with corresponding HCM presentations (Figure 1 and Figure 2).

## 4. Discussion

To the best of our knowledge, this is the first study of shared molecular mechanisms of HCM and its clinical presentations.

Although there is much research about molecular mechanisms of HCM, research about molecular mechanisms in its specific clinical presentations is scarce. In the following literature review, we compared our results with evidence from preclinical and clinical literature.

### 4.1. Shared Molecular Elements and Pathways

#### 4.1.1. Hypertrophic Cardiomyopathy and Structural Changes

The most important shared mechanisms of cardiomyocyte hypertrophy and HCM are those involved in fibrosis, calcium, and energy homeostasis. ET-1 strongly induces cardiomyocyte hypertrophy in HCM-induced pluripotent stem-cells-derived cardiomyocytes, with ET-1 stimulation specifically inducing NFAT nuclear accumulation [39]. Angiotensin II induces cardiomyocyte hypertrophy [40,41,42,43].

The most important shared molecular mechanisms of myofibrillar disarray and HCM are contractile machinery components and those involved in ATP homeostasis, MAPK/ERK, and calcineurin/NFAT signaling. Frustaci et al. (2018) showed that in humans, mutation of sarcomeric α-actin is followed by fibrils disarray and hypertrophy with a disarray of cardiomyocytes, while dysfunction of cytoplasmic α-actin causes a disanchorage of myofibrils from the sarcolemmal membrane, followed by myofibrillolysis. The authors proposed that intercalated discs are particularly involved in this mutation, appearing irregular and fragmented, favoring cell disconnection [44]. Tanaka et al. (2014) showed that endothelin-1 induces myofibrillar disarray in HCM-induced pluripotent stem-cell-derived cardiomyocytes [39].

Our results indicate that the contractile machinery components and mechanisms involved in cell growth and survival are the most prominent mutual molecules and processes involved in cardiomyocyte disarray and HCM. Kraft et al. (2019) suggested that mutations in *MYH7* in heterozygous human HCM contribute to the development of cardiomyocyte disarray by burst-like heterogeneous expressions of both *MYH7* alleles (switched on and off in an independent and stochastic manner), which causes an imbalanced force generation going from cell to cell that disrupts the cardiac syncytium over time (stronger cells overstretch weaker cells) [45]. Schramm et al. (2012) showed that the *PTPN11* loss-of-function mutation Q510E-Shp2 causes cardiomyocyte disarray in HCM, with mTOR activation playing a critical role in the underlying mechanism [46]. James et al. (2000) demonstrated that one of the manifestations of cTnI^146Gly^ mutation in mice is cardiomyocyte disarray [47].

Our results indicate that calcium homeostasis, fibrosis, and inflammation mechanisms are the most important at the intersection of myocardial remodeling and HCM.

The most important shared elements of cardiac remodeling and HCM are implicated in fibrosis, calcium, and energy homeostasis. Freeman et al. (2001) showed that a high overexpression of β2-adrenergic receptor increases remodeling in HCM hearts and that inhibition of β-adrenergic receptor kinase (βARK) reverses hypertrophic remodeling in the HCM hearts [48]. Martins et al. (2015) suggested that the TNNC1-A8V mutant increases the calcium-binding affinity of the thin filament and elicits cellular remodeling [49]. Bi et al. (2021) showed that collagen cross-linking plays an important role in heart remodeling in human hypertrophic obstructive cardiomyopathy, which might be regulated mainly by lysyl oxidase (LOX) [50]. Roldán et al. (2008) suggested that the matrix metalloproteinases have an important role in cardiac remodeling in human HCM [51].

Shared molecules of myocardial fibrosis and HCM are those entangled in calcium and energy homeostasis, fibrosis, cell survival, proliferation, and inflammation. Ho et al. (2010) suggested that, in human HCM, sarcomere mutations trigger an early increase in collagen synthesis; this is initially balanced by degradation, but it exceeds degradation in overt HCM synthesis, resulting in myocardial fibrosis (i.e., collagen accumulation in HCM increases as the disease develops) [52]. Kawano et al. (2005) showed that valsartan (an angiotensin II type 1 receptor blocker) suppresses the synthesis of type I collagen in patients with HCM [53]. Arteaga et al. (2009) showed that myocardial fibrosis is prospectively associated with a worse prognosis in patients with HCM [54]. Further, Lim et al. (2001) showed that the blockade of angiotensin II (a known cardiotrophic factor) by losartan reverses myocardial fibrosis in a transgenic mouse model of human HCM [55].

#### 4.1.2. Hypertrophic Cardiomyopathy and Left Ventricular Outflow Tract Obstruction

Scarce molecular mechanisms found at the intersection of LVOTO and HCM are indicative of an important role in calcium homeostasis and fibrosis. Bolca et al. (2002) showed that dobutamine induces dynamic LVOTO in patients with hypertrophic non-obstructive cardiomyopathy, proving that dobutamine stress echocardiography is a reliable tool for the diagnosis of dynamic left ventricular obstruction in patients with hypertrophic non-obstructive cardiomyopathy [56]. Mavacamten (a first-in-class cardiac myosin inhibitor) has been evaluated as a promising new therapy in several clinical studies [57,58,59].

#### 4.1.3. Hypertrophic Cardiomyopathy and Contractile Dysfunction

Although the molecular mechanisms found at the intersection of impaired myocardial relaxation and HCM are scarce, they indicate the leading role of NO homeostasis and a contribution of calcium homeostasis and collagen induction in their common pathogenesis. Cordts et al. (2019) suggested that higher N, N-dimethylarginine (also known as asymmetric dimethylarginine, ADMA) plasma concentrations might lead to a decreased NO production and an impaired myocardial relaxation in HCM patients [24].

Titin and titin-related molecules were found to be important in the intersection of myocardial stiffness and HCM. Higashikuse et al. (2019) suggested that titin mutations in HCM families can be incorporated into the sarcomere and impair TRIM63 (MURF1) binding, resulting in abnormal sarcomere stiffness [60].

Our results indicate that the contractile machinery components and mechanisms involved in calcium, sodium, and cellular energy homeostasis are the most prominent common molecules of diastolic dysfunction and HCM. Diastolic dysfunction in animal and human HCM is characterized by elevated myocardial activation at low diastolic calcium concentrations, i.e., high myofilament calcium-sensitivity [61,62,63,64]. In the majority of cases, the high basal (diastolic) myofilament activation is sufficient to slow the onset of ventricular relaxation and limit proper filling [62]. Sequeira et al. (2015) showed that tropomyosin’s ability to block myosin-binding sites on actin is reduced in human HCM with thin-filament mutations, and the effect is exacerbated in human HCM samples by the low PKA phosphorylation of myofilament proteins. They also suggested that cMyBP-C HCM-causing mutations reduce the accessibility of myosin for actin [65]. Teekakirikul et al. (2010) suggested that TGF-β signaling is implicated in progressive diastolic dysfunction in HCM [66]. Dweck et al. (2014) suggested that the inability to enhance myofilament relaxation through cardiac troponin I phosphorylation predisposes the heart to abnormal diastolic function [67]. Alves et al. (2015) proposed that troponin I may be an important target for the development of myofilament calcium desensitizers [68]. Further, Granzier et al. (2009) showed that the absence of PEVK region (one of the two major elastic elements of cardiac titin molecule) results in diastolic dysfunction [69].

#### 4.1.4. Hypertrophic Cardiomyopathy and Arrhythmia

Our results suggest that the most important common mechanisms of AF and HCM are calcium and sodium homeostasis in cardiomyocytes. Bongini et al. (2016) suggested that RyR2 malfunction (probably by spontaneous sarcoplasmic reticulum calcium leakage) might represent a general pathophysiologic mechanism for AF initiation and maintenance in human HCM [70]. Nagai et al. (2007) found a significant association between the prevalence of AF and ACE polymorphism in patients with HCM [71].

Our results suggest that the most important shared molecular elements of SCD and HCM are contractile machinery components as well as sodium, calcium, and energy homeostasis mechanisms. Okuda et al. (2018) proved that CaMKII-mediated phosphorylation of RyR2 plays a crucial role in aberrant calcium release as a potent substrate of lethal arrhythmia in HCM-linked Troponin T-mutated hearts [72]. Alterations in calcium cycling are triggers for cardiac arrhythmias—a serious clinical complication of HCM due to the potential to induce SCD [73]. On the other hand, calcium may be involved in the development of cardiac fibrosis, a potential substrate for cardiac arrhythmias and sudden death. In humans, mutations of calcium-related genes (RyR2 and calsequestrin 2) have been identified in families with a history of SCD [74]. Studies with HCM cardiomyocytes differentiated from patient-specific-induced pluripotent stem cells have confirmed that alterations of intracellular calcium handling are associated with arrhythmic events [75]. Coppini et al. (2020) suggested that increased late sodium current (I_NaL_) plays a central role in cellular arrhythmogenicity in HCM (which is confirmed by the antiarrhythmic efficacy of ranolazine) [76]. Parvatiyar et al. (2012) showed that *TNNC1* mutation A31S, which alters calcium handling, is associated with verified episodes of ventricular fibrillation and aborted SCD, probably due to altered calcium handling and electrophysiologic remodeling of the cardiomyocyte [77]. Additionally, Chung et al. (2011) found that frameshift mutation (c.363dupG) in Troponin C is associated with HCM and SCD [78]. Further, Fahed et al. (2020) showed that p.Arg21Cys mutation in *TNNI3* impairs calcium handling and results in a malignant HCM phenotype characterized by early-onset SCD [79]. HCM caused by mutations in the cardiac troponin T gene (*TNNT2*) has been associated with a high risk of SCD [80]. R58Q mutation of myosin regulatory light chain (*RLC*) is associated with SCD in HCM [81].

#### 4.1.5. Hypertrophic Cardiomyopathy and Ischemia

We found only several common mechanisms in both coronary microvascular dysfunction and HCM. However, they showed the greatest importance of energy, calcium, and sodium homeostasis in the intersection of these two pathologies. We suggest that extracted interaction “glucose is increased/activated by insulin” refers to insulin-dependent glucose transport into cells, and “calcium increased/activated by calcium” is related to calcium-induced calcium release [82].

The most important elements of the intersection of molecular mechanisms of myocardial ischemia and HCM take part in calcium, sodium, and energy homeostasis, ERK signaling, inflammation, and cell survival.

#### 4.1.6. Hypertrophic Cardiomyopathy and Endpoints

The most important shared molecular elements of HF and HCM are calcium, sodium, and energy homeostasis mechanisms, contractile machinery components, ERK signaling, β-adrenergic receptor mechanisms, and those entangled in fibrosis, cell proliferation, and survival. Mutations of *MYBPC3* gene are a major cause of human cardiomyopathy and associated HF [83]. *MYBPC3* mutations present a high risk for HF [84]. Kissopoulou et al. (2018) showed that homozygous missense *MYBPC3* Pro873His mutation in human HCM is associated with an increased risk of HF development [85]. Chronic administration of β-adrenergic agonists, such as isoproterenol, has been shown to aggravate HCM and induce HF in HCM models of disease [86].

We could not abstract the essence of the intersections of MACE and HCM or rehospitalization and HCM from the corresponding heterogeneous results, probably on account of the diverse pathologies underlying both MACE and rehospitalization.

#### 4.1.7. Calcium in Hypertrophic Cardiomyopathy Presentations

Our results suggest that calcium is among the most important elements in almost all intersections of molecular pathways of HCM and its clinical presentations. Calcium is a key signaling molecule in the cardiac myocyte [74], and imbalances in calcium homeostasis have been described as key characteristics of HCM in numerous reports [73].

#### 4.1.8. The Most Important Shared Elements and Pathways

As expected, at a high level, our results show that cardiomyocyte hypertrophy, myocardial and cardiac remodeling, and myocardial fibrosis; AF and SCD; coronary microvascular dysfunction and myocardial ischemia; myocardial ischemia and HF share similar molecular mechanisms, which is in line with clinical literature findings on HCM progression [87,88], arrhythmic nature and association between AF and SCD [89,90,91], ischemic nature and association between coronary microvascular dysfunction and myocardial ischemia [25,92,93], and association between myocardial ischemia and HF in HCM [94,95]. The results suggest a more isolated (distinctive) nature of myofibrillar and cardiomyocyte disarray, impaired myocardial relaxation, and myocardial stiffness, which might be, to some extent, a consequence of the relatively low number of articles available and statements extracted, which then reduce the ability to identify the most important molecular elements.

### 4.2. Non-Molecular Factors That Affect Clinical Presentations of HCM

Phenotypes of HCM are the consequences of complex interactions among a large number of determinants [96]. In addition to molecular mechanisms (including genetic factors), other factors can affect the clinical course and presentations of HCM. Environmental and lifestyle factors, most probably via epigenetic mechanisms, influence HCM phenotype [97,98,99]. These factors and their interaction in HCM have yet to be fully defined but might include microbial infection, diet [97], or exercise [96,97,98]. A study by Repetti et al. suggested that epigenetic and environmental factors, rather than background genetic variation, play a major role in hypertrophic remodeling [97].

Incomplete penetrance [96] and haploinsufficiency [84,99] also complicate interpretations of genotype-phenotype associations [96] and the prediction of clinical presentations. Phenotypic effects in cases of incomplete penetrance are even more responsive to the presence of other genetic and environmental factors. Cell-to-cell variability in gene expression and function also affect the HCM phenotype [96].

Physical factors like pressure changes, stretching, and changes in the generation of contraction force also influence the clinical course of HCM [96].

Other known and unknown factors might contribute to the development of different HCM clinical presentations as well.

By that means, this research, in its broad scope, is interesting for providing the potential of identification of molecular targets for environmental factors or lifestyle choices that could delay or change HCM progression.

### 4.3. General

All patients with HCM defined according to ESC guidelines [92] were included. No uniform exclusion criteria were applied.

Many molecular elements recognized as important in this research are non-specific and take part in different cardiac processes and diseases. Some of them might be compensatory mechanisms.

With the approach undertaken in the present study, we were able to detect shared mechanisms that might otherwise remain unnoticed. Although we cannot state that shared mechanisms determine or underlie the clinical presentation of HCM, these shared mechanisms have the potential to direct HCM processes or modify the nature of each disease state. Some of them might be novel therapeutic targets or contribute to the development of innovative strategies for treatment. This research also provides the potential to identify patients with specific or non-specific HCM molecular milieu patterns and with that preventability of certain complications or predisposition to side effects.

In silico studies of molecular interactions rarely provide final answers to questions. Nevertheless, very often, they produce a foundation for further research and initialize the generation of new questions and hypotheses. This work represents only the first step in the dissection of HCM pathogenesis, which could inspire and intensify future research. These results should be used after careful interpretation and critical evaluation of each element of interest in a particular use case.

Thus far, INDRA and the automated extraction of molecular mechanisms have been used in modeling p53 dynamics in response to DNA damage, adaptive drug resistance in BRAF-V600E-mutant melanomas, and the RAS signaling pathway [27].

Based on the literature review, the method applied has the potential to be beneficial in similar use cases. However, there is space for improvement of the technology and its implementation.

### 4.4. Limitations

The number of elements in networks (and sets) reflects the quantum of knowledge published on the topic rather than the complexity or granularity of the mechanisms themselves.

The automatic molecular mechanisms extraction approach is not specific (it extracts all molecular interactions from the article with the particular main topic), which is why each interaction should be considered critically.

The automatic extraction of molecular mechanisms sometimes extracts gene products with the name of the corresponding genes. Therefore, when evaluating an element with a gene name, it must be interpreted as the gene itself and/or its product.

Although automated extraction of molecular mechanisms creates a lot of clutter (e.g., elements not representing molecular mechanisms), we suppose that the nature of intersection removes most of the clutter (i.e., a piece of clutter should be present in two intersected sets to appear in results).

Both preclinical and clinical articles were included in the automatic molecular mechanisms extraction. Animal models do not fully replicate human HCM [100]. Our research lacks overall comorbidity information (it is source-article-specific).

### 4.5. Significance and Implications

This work collects and represents a quantum of knowledge about shared molecular mechanisms of HCM and its clinical presentations available today. Our results do not represent the final nor perfect dissection of HCM pathogenesis, yet they offer a transitional solution towards the next step in the research on HCM and its clinical presentations. It represents a wide foundation for further research, where new starting points could be found.

All pathways are presented in visual, and by that more intuitive, form, in one place. This work can be seen as a detailed review on the topic in the form of networks (instead of in the form of text) generated automatically (instead of by systematic literature inspection and writing). The pathways in the form of networks enable further analysis, for example, for in silico screening of new biomarkers and drug targets, as well as for predicting additional missing links and elements.

Shared pathways are commonly researched using different approaches [101,102,103,104,105,106,107,108,109,110,111,112,113,114]. The novelty in shared pathways research is the application of the new technology, automated molecular mechanisms extraction, to that task. In this research, we were also examining the reach of the technology used for automated extraction of molecular mechanisms from scientific medical literature. This approach is new in deciphering molecular mechanisms of HCM. Some parts of the methodology are taken over from the big data analysis field [14], and this research is one of the first attempts to analyze such massive data in the domain of this specific clinical entity.

This research also confirms that the results of usage of the technology are consistent with the information present in the scientific literature at a higher level, but also that there is a space for improvement of the technology and its implementation.

## 5. Conclusions

The most important molecular mechanisms that HCM shares with its clinical presentations are as follows: fibrosis, calcium and energy homeostasis (shared with cardiomyocyte hypertrophy and cardiac remodeling); contractile machinery components, ATP homeostasis, MAPK/ERK, and calcineurin/NFAT signaling (myofibrillar disarray); contractile machinery components and mechanisms involved in cell growth and survival (cardiomyocyte disarray); calcium homeostasis, fibrosis and inflammation mechanisms (myocardial remodeling); calcium and energy homeostasis, fibrosis, cell survival, proliferation and inflammation (myocardial fibrosis); calcium homeostasis and fibrosis (LVOTO); NO and calcium homeostasis, collagen induction (impaired myocardial relaxation); titin and titin-related molecules (myocardial stiffness); calcium and sodium homeostasis in cardiomyocytes (AF); contractile machinery components and mechanisms involved in calcium, sodium, and energy homeostasis (SCD and diastolic dysfunction); energy, calcium and sodium homeostasis mechanisms (coronary microvascular dysfunction); calcium, sodium and energy homeostasis; ERK signaling, inflammation and cell survival mechanisms (myocardial ischemia); calcium, sodium, and energy homeostasis mechanisms; contractile machinery components; ERK signaling; β-adrenergic receptor mechanisms; mechanisms entangled in fibrosis, cell proliferation and survival (HF). These mechanisms represent possible processes underlying different HCM clinical presentations, and some of them might be novel therapeutic targets.

This work collects and represents a quantum of knowledge about shared molecular mechanisms of HCM and its clinical presentations available today.

Applied methodology produced results consistent with the information in the scientific literature at a higher level, but there is a space for improvement of the technology and its implementation.

## Figures and Tables

**Table 1:** The number of articles on hypertrophic cardiomyopathy and its clinical presentations read automatically and the number of INDRA statements extracted.

Pathophysiologic Entity	Number of Articles Read Automatically	Number of INDRA Statements Extracted
hypertrophic cardiomyopathy	8111	7559
cardiomyocyte hypertrophy	1337	2500
myofibrillar disarray	51	356
cardiomyocyte disarray	11	22
myocardial remodeling	967	1500
cardiac remodeling	4572	5432
myocardial fibrosis	3634	4978
left ventricular outflow tract obstruction	1023	177
myocardial hypercontractility	3	3
impaired myocardial relaxation	31	33
impaired cardiac relaxation	12	28
myocardial stiffness	257	500
diastolic dysfunction	6342	6101
atrial fibrillation	54,117	25,842
sudden cardiac death	10,060	6770
coronary microvascular dysfunction	569	522
myocardial ischemia	19,637	19,078
heart failure	111,565	98,397
major adverse cardiovascular events	4700	1713
rehospitalization	3073	656

**Table 2:** Shared molecular mechanisms of hypertrophic cardiomyopathy and its clinical presentations.

Pathophysiologic Entities	Link to the Network Representing Shared Molecular Mechanisms
hypertrophic cardiomyopathy, cardiomyocyte hypertrophy	https://bit.ly/39Yn90x (accessed on 1 August 2021)
hypertrophic cardiomyopathy, myofibrillar disarray	https://bit.ly/2PRnPOz (accessed on 1 August 2021)
hypertrophic cardiomyopathy, cardiomyocyte disarray	https://bit.ly/3wJsmmy (accessed on 1 August 2021)
hypertrophic cardiomyopathy, myocardial remodeling	https://bit.ly/2Q8dDkD (accessed on 1 August 2021)
hypertrophic cardiomyopathy, cardiac remodeling	https://bit.ly/31ZG3Qh (accessed on 1 August 2021)
hypertrophic cardiomyopathy, myocardial fibrosis	https://bit.ly/3fZX3hC (accessed on 1 August 2021)
hypertrophic cardiomyopathy, left ventricular outflow tract obstruction	https://bit.ly/3dN8G8R (accessed on 1 August 2021)
hypertrophic cardiomyopathy, impaired myocardial relaxation	https://bit.ly/322sU94 (accessed on 1 August 2021)
hypertrophic cardiomyopathy, myocardial stiffness	https://bit.ly/3mxmecq (accessed on 1 August 2021)
hypertrophic cardiomyopathy, diastolic dysfunction	https://bit.ly/3wHxRCn (accessed on 1 August 2021)
hypertrophic cardiomyopathy, atrial fibrillation	https://bit.ly/3d31kyT (accessed on 1 August 2021)
hypertrophic cardiomyopathy, sudden cardiac death	https://bit.ly/3wIN5ao (accessed on 1 August 2021)
hypertrophic cardiomyopathy, coronary microvascular dysfunction	https://bit.ly/31Xh2VN (accessed on 1 August 2021)
hypertrophic cardiomyopathy, myocardial ischemia	https://bit.ly/31YlC6a (accessed on 1 August 2021)
hypertrophic cardiomyopathy, heart failure	https://bit.ly/322UjI9 (accessed on 1 August 2021)
hypertrophic cardiomyopathy, major adverse cardiovascular events	https://bit.ly/3mvZZE1 (accessed on 1 August 2021)
hypertrophic cardiomyopathy, rehospitalization	https://bit.ly/3myyx8t (accessed on 1 August 2021)

**Table 3:** Top nodes of each network ranked by centrality scores.

Network	Top Nodes Ranked by Centrality Scores
hypertrophic cardiomyopathy, cardiomyocyte hypertrophy	https://bit.ly/3fCs1Mq (accessed on 1 August 2021)
hypertrophic cardiomyopathy, myofibrillar disarray	https://bit.ly/2OgKHpM (accessed on 1 August 2021)
hypertrophic cardiomyopathy, cardiomyocyte disarray	https://bit.ly/31LLUsi (accessed on 1 August 2021)
hypertrophic cardiomyopathy, myocardial remodeling	https://bit.ly/3uj130t (accessed on 1 August 2021)
hypertrophic cardiomyopathy, cardiac remodeling	https://bit.ly/39CYWgj (accessed on 1 August 2021)
hypertrophic cardiomyopathy, myocardial fibrosis	https://bit.ly/3dc8HUA (accessed on 1 August 2021)
hypertrophic cardiomyopathy, left ventricular outflow tract obstruction	https://bit.ly/3cJRWzY (accessed on 1 August 2021)
hypertrophic cardiomyopathy, impaired myocardial relaxation	https://bit.ly/3dubAz7 (accessed on 1 August 2021)
hypertrophic cardiomyopathy, myocardial stiffness	https://bit.ly/2PpsZRM (accessed on 1 August 2021)
hypertrophic cardiomyopathy, diastolic dysfunction	https://bit.ly/2PQuwju (accessed on 1 August 2021)
hypertrophic cardiomyopathy, atrial fibrillation	https://bit.ly/2OhvNzE (accessed on 1 August 2021)
hypertrophic cardiomyopathy, sudden cardiac death	https://bit.ly/3ugy2CI (accessed on 1 August 2021)
hypertrophic cardiomyopathy, coronary microvascular dysfunction	https://bit.ly/3wiA5YR (accessed on 1 August 2021)
hypertrophic cardiomyopathy, myocardial ischemia	https://bit.ly/39Hexvk (accessed on 1 August 2021)
hypertrophic cardiomyopathy, heart failure	https://bit.ly/3uwQiYP (accessed on 1 August 2021)
hypertrophic cardiomyopathy, major adverse cardiovascular events	https://bit.ly/3fHsE7w (accessed on 1 August 2021)
hypertrophic cardiomyopathy, rehospitalization	https://bit.ly/3dzxLUy (accessed on 1 August 2021)

**Table 4:** Networks with different PE-values applied. PE-measure (the measure for interaction reliability) removes spurious interactions (below the value applied) and, thus, the level of noise in networks.

Network	Link to Networks with Different PE-Values Applied
hypertrophic cardiomyopathy, cardiomyocyte hypertrophy	https://bit.ly/3sMXLm1 (accessed on 1 August 2021)
hypertrophic cardiomyopathy, myofibrillar disarray	https://bit.ly/39Ebt2N (accessed on 1 August 2021)
hypertrophic cardiomyopathy, cardiomyocyte disarray	https://bit.ly/3cMpoGd (accessed on 1 August 2021)
hypertrophic cardiomyopathy, myocardial remodeling	https://bit.ly/3duSh8V (accessed on 1 August 2021)
hypertrophic cardiomyopathy, cardiac remodeling	https://bit.ly/3dBWPdU (accessed on 1 August 2021)
hypertrophic cardiomyopathy, myocardial fibrosis	https://bit.ly/324nVoj (accessed on 1 August 2021)
hypertrophic cardiomyopathy, left ventricular outflow tract obstruction	https://bit.ly/31GacUC (accessed on 1 August 2021)
hypertrophic cardiomyopathy, impaired myocardial relaxation	https://bit.ly/31MjKxv (accessed on 1 August 2021)
hypertrophic cardiomyopathy, myocardial stiffness	https://bit.ly/3sMZnfz (accessed on 1 August 2021)
hypertrophic cardiomyopathy, diastolic dysfunction	https://bit.ly/3cNFNu8 (accessed on 1 August 2021)
hypertrophic cardiomyopathy, atrial fibrillation	https://bit.ly/3mhXtRv (accessed on 1 August 2021)
hypertrophic cardiomyopathy, sudden cardiac death	https://bit.ly/3wxjGzZ (accessed on 1 August 2021)
hypertrophic cardiomyopathy, coronary microvascular dysfunction	https://bit.ly/3uhQQ4l (accessed on 1 August 2021)
hypertrophic cardiomyopathy, myocardial ischemia	https://bit.ly/31GmOLu (accessed on 1 August 2021)
hypertrophic cardiomyopathy, heart failure	https://bit.ly/3mi5i9U (accessed on 1 August 2021)
hypertrophic cardiomyopathy, major adverse cardiovascular events	https://bit.ly/2QZt66N (accessed on 1 August 2021)
hypertrophic cardiomyopathy, rehospitalization	https://bit.ly/2QYjedr (accessed on 1 August 2021)

## Data Availability

Data are contained within the article and supplementary material.

## Supplementary Materials

The following are available online at https://www.mdpi.com/article/10.3390/life11080785/s1, Supplementary Table S1: The most important nodes in the networks, Table S2: Cooperatively working elements (functional modules), Figure S1: The most important nodes in networks represented as packed concentric rings sorted by the most important nodes.
