# Supplementary material for: Shared Molecular Mechanisms of Hypertrophic Cardiomyopathy and Its Clinical Presentations: Automated Molecular Mechanisms Extraction Approach
Source: Life (Basel). 2021 Aug 3;11(8):785. doi: 10.3390/life11080785 (PMC8398249; doi:10.3390/life11080785)
Supplement: Supplementary file 1 [file life-11-00785-s001.zip › Figure S1.pdf]

Hypertrophic cardiomyopathy ∩ cardiomyocyte hypertrophy

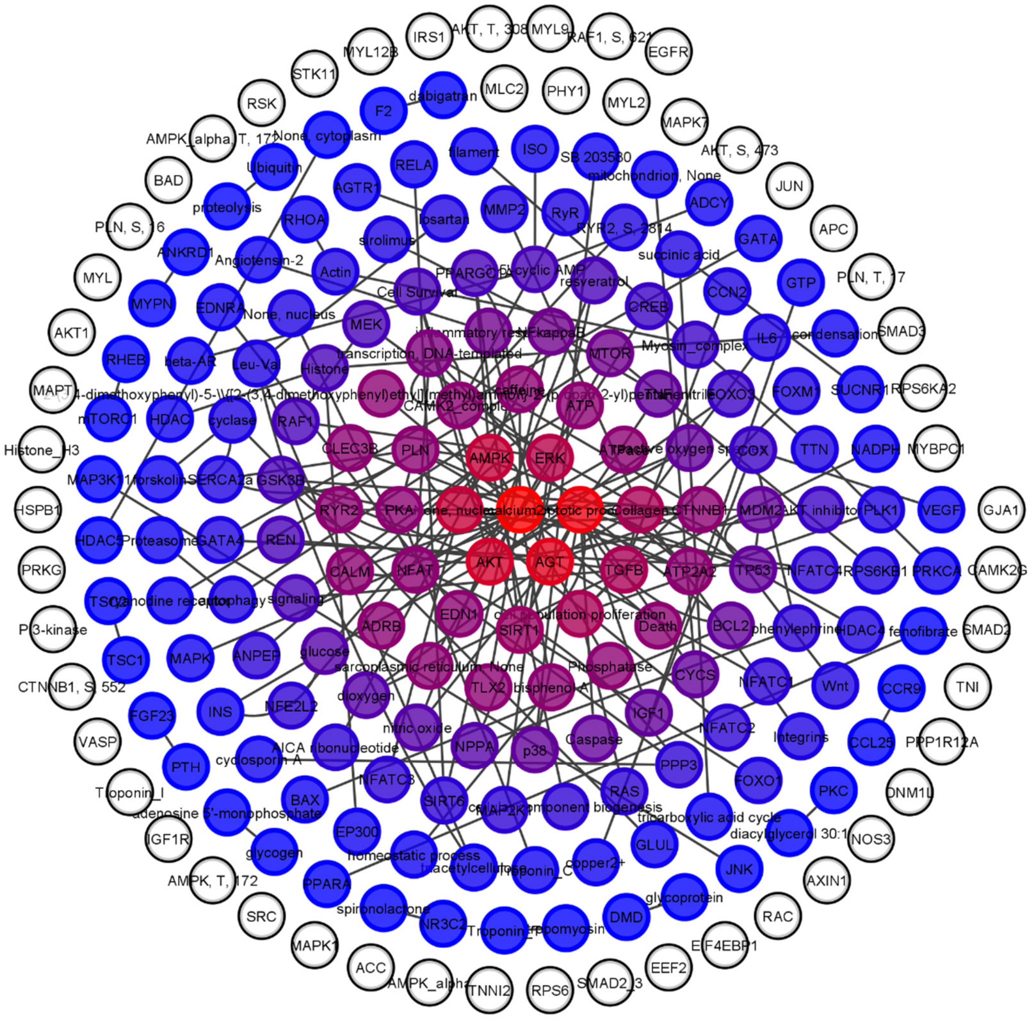

Hypertrophic cardiomyopathy ∩ myofibrillar disarray

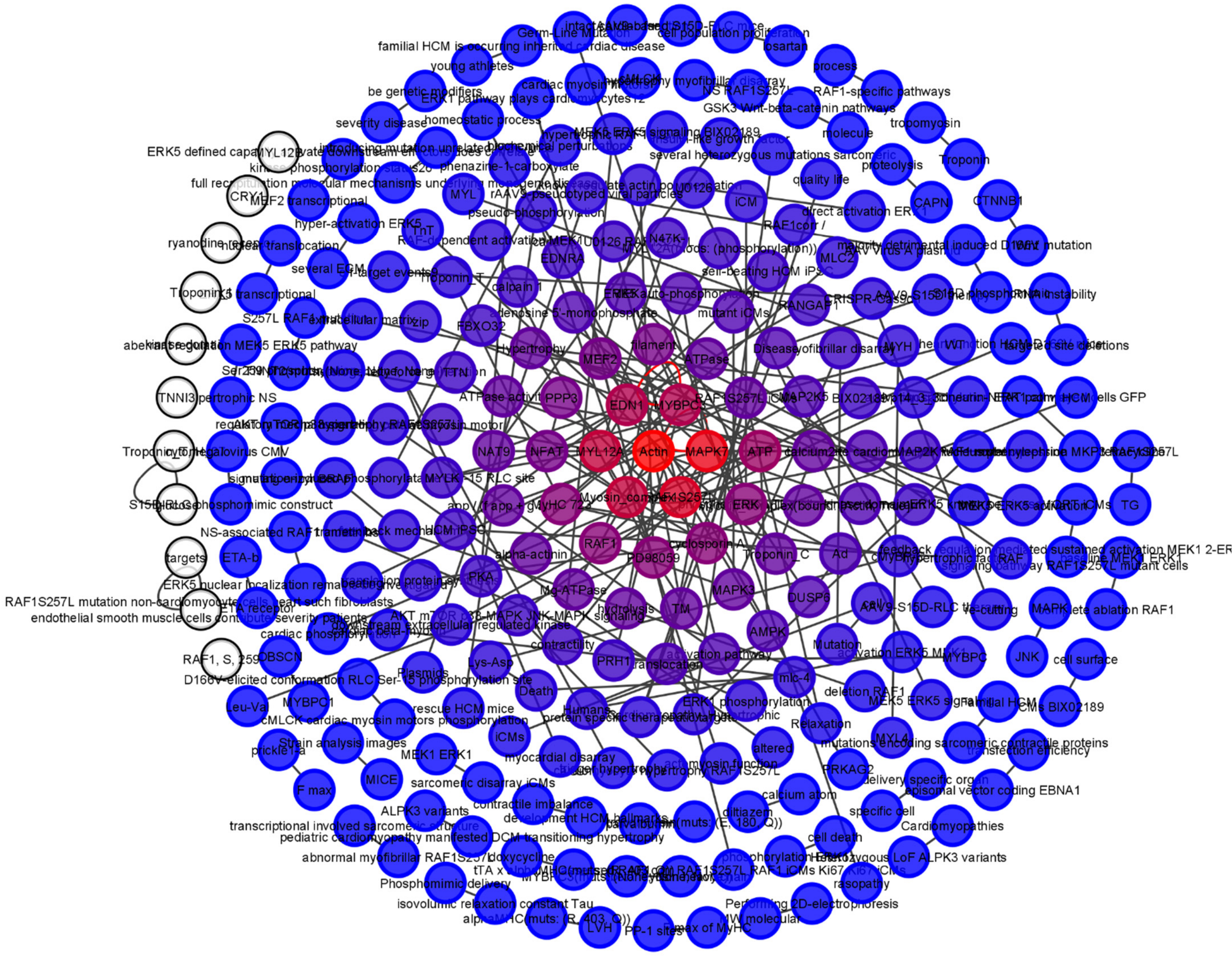

Hypertrophic cardiomyopathy ∩ cardiomyocyte disarray

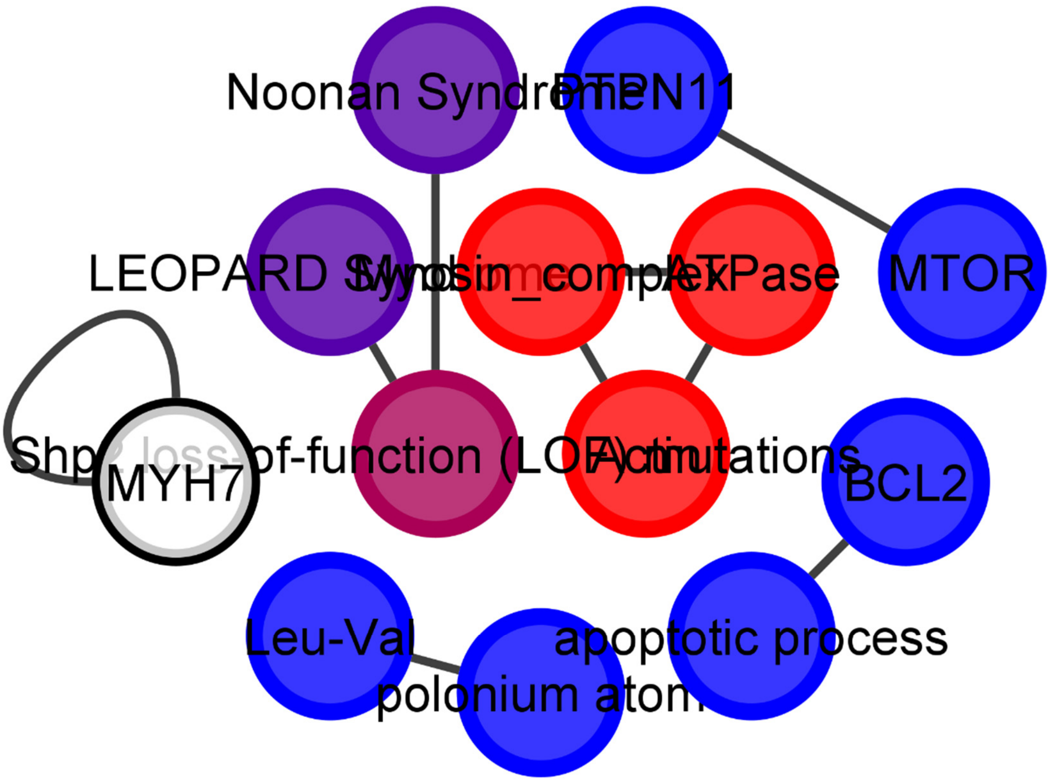

Hypertrophic cardiomyopathy ∩ myocardial remodeling

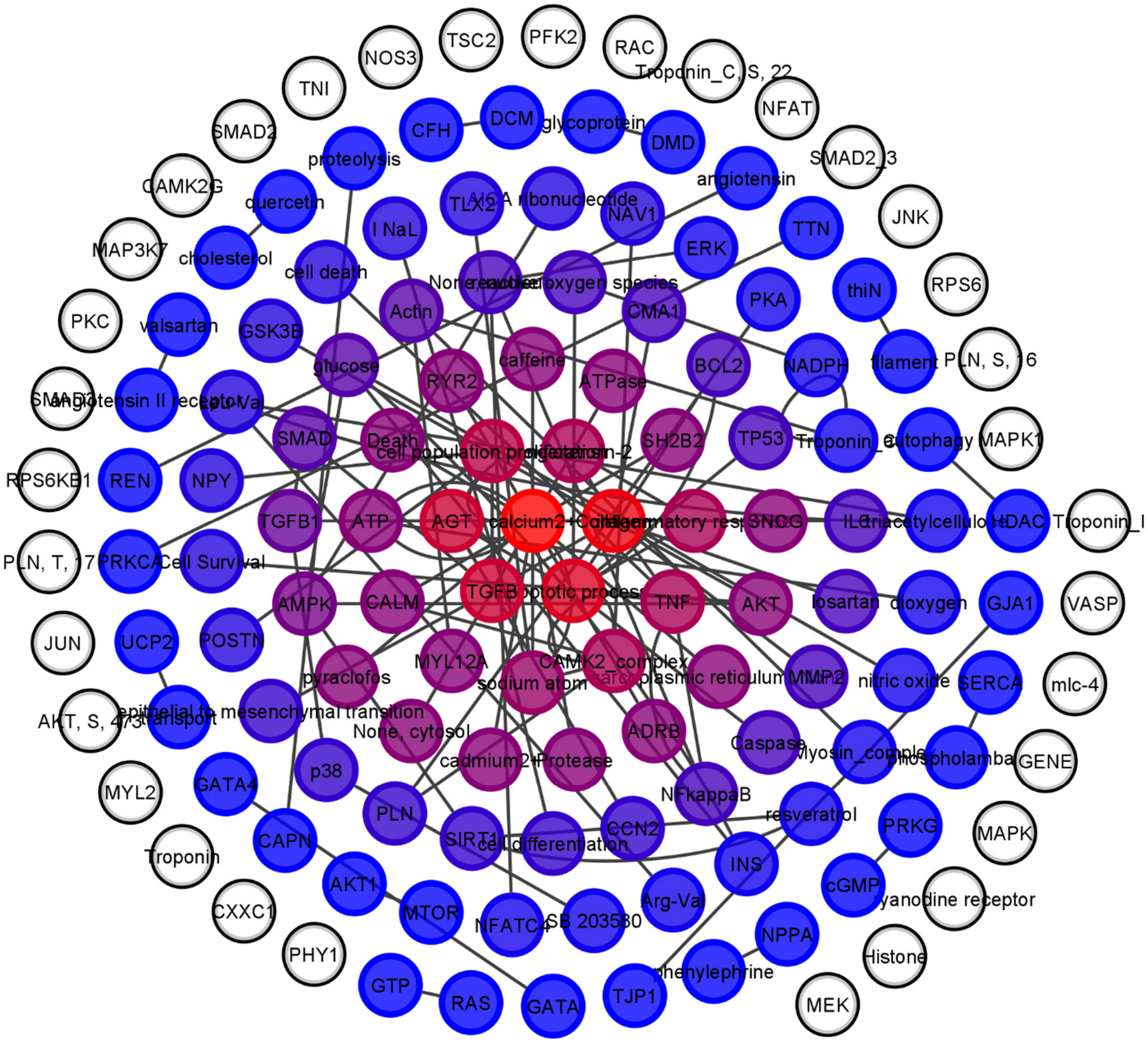

Hypertrophic cardiomyopathy ∩ cardiac remodeling

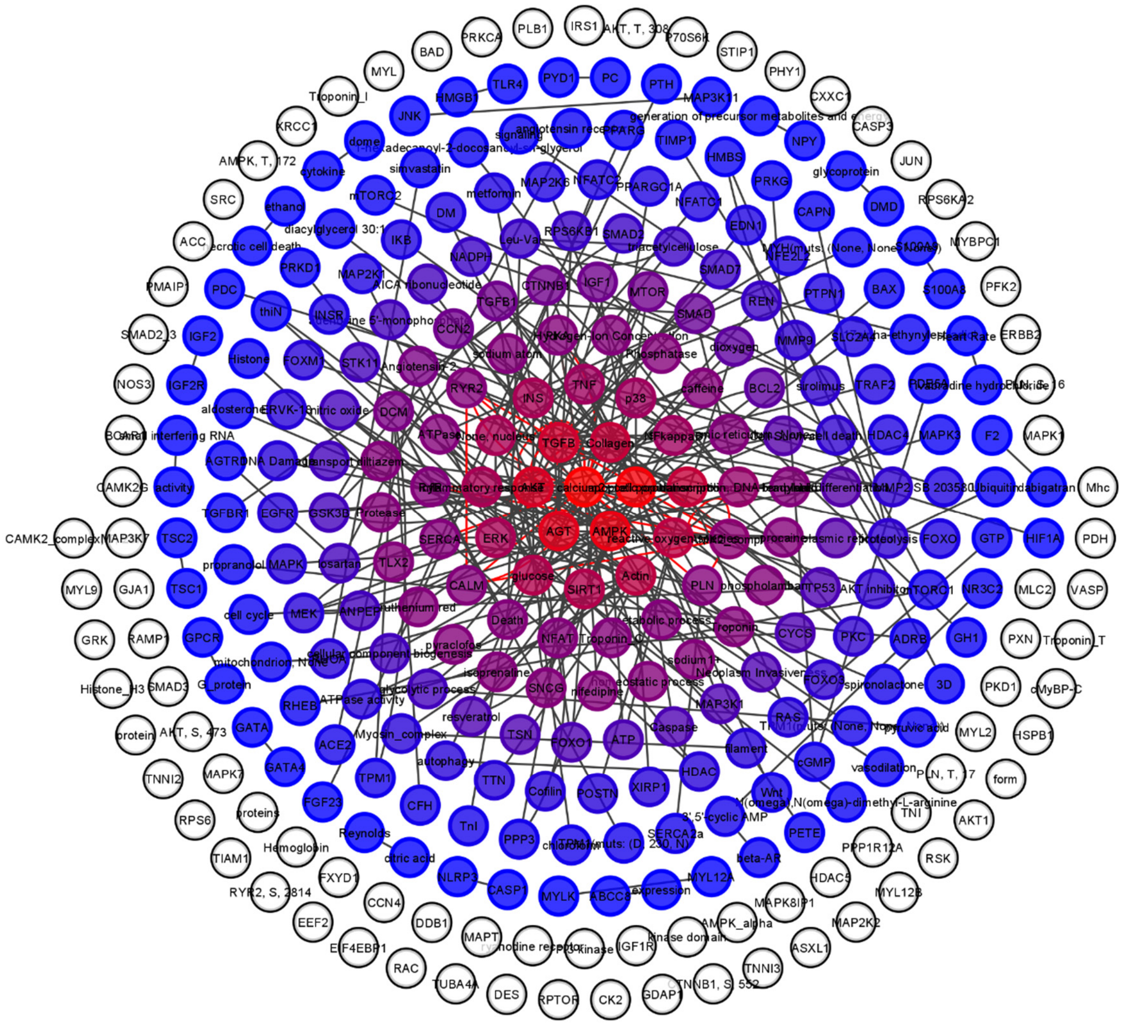

Hypertrophic cardiomyopathy ∩ myocardial fibrosis

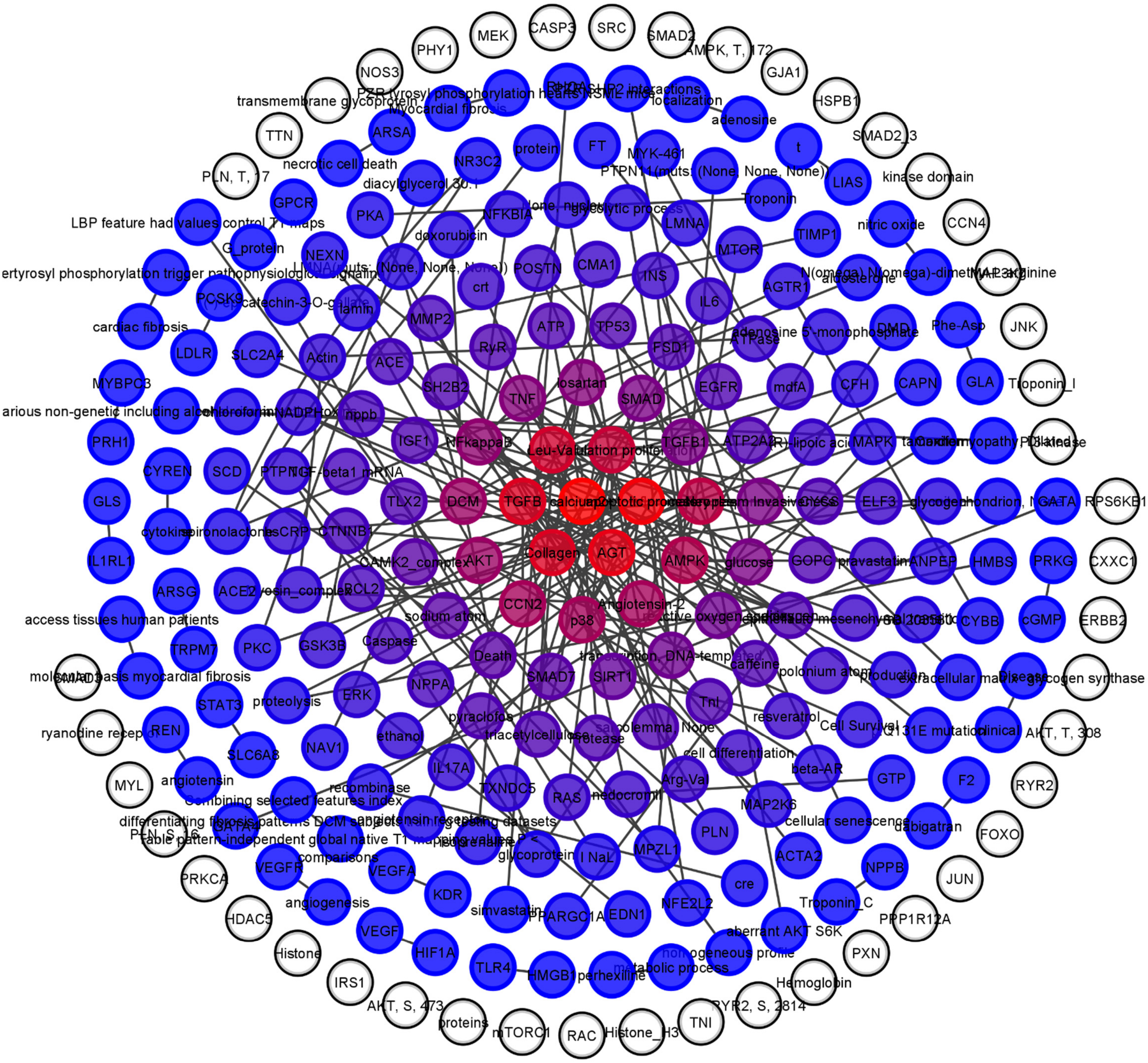

Hypertrophic cardiomyopathy ∩ left ventricular outflow tract obstruction

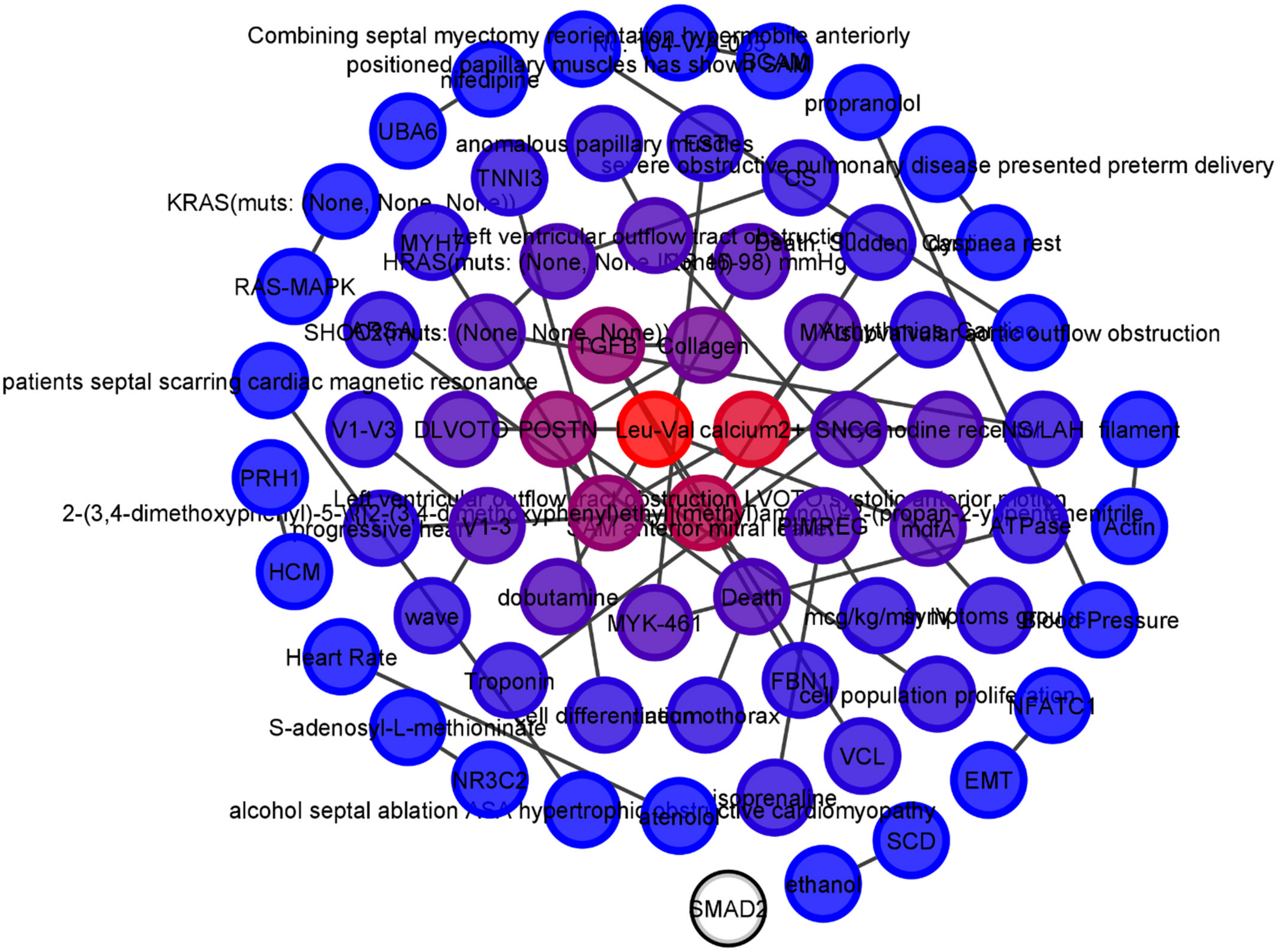

Hypertrophic cardiomyopathy ∩ impaired myocardial relaxation

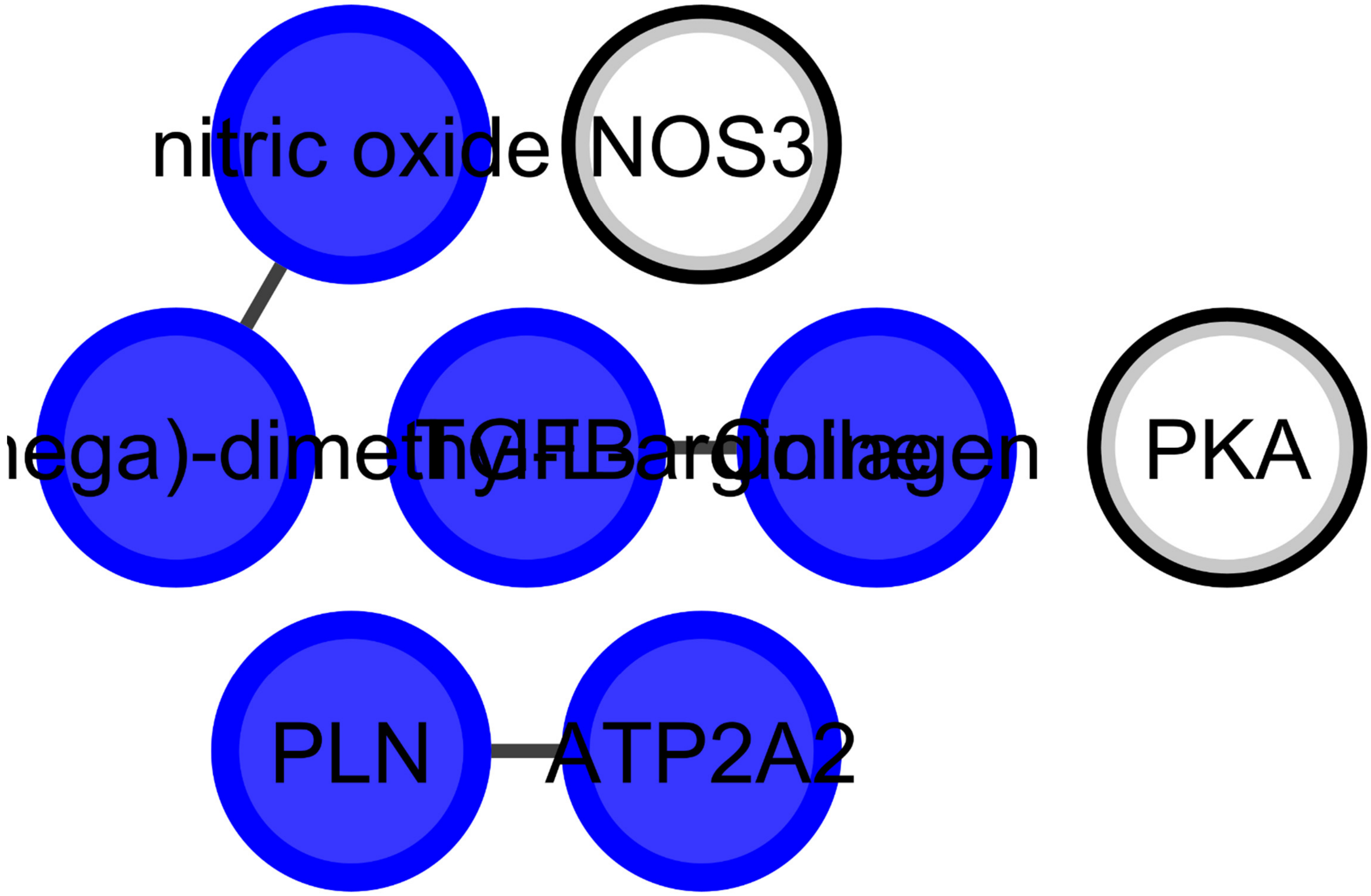

Hypertrophic cardiomyopathy ∩ myocardial stiffness

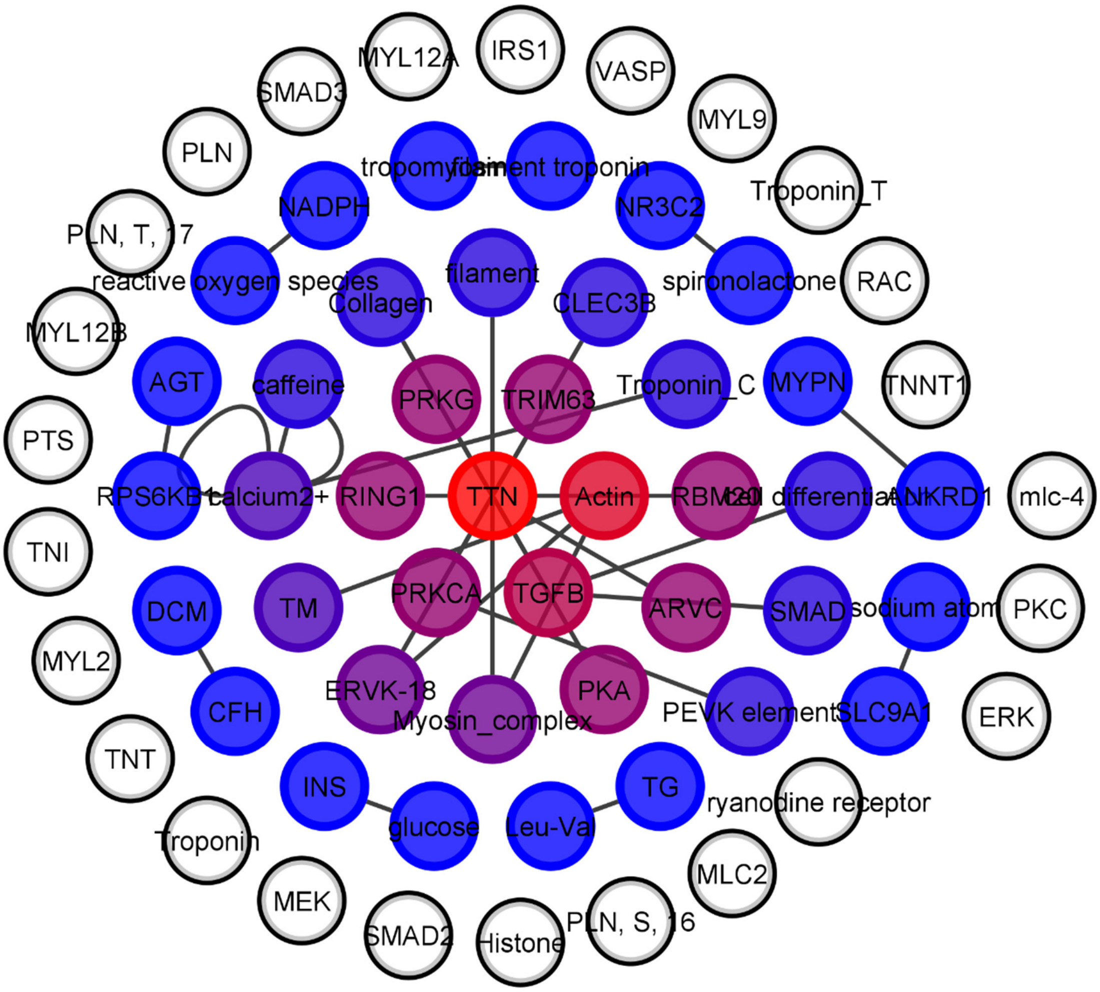

Hypertrophic cardiomyopathy ∩ diastolic dysfunction

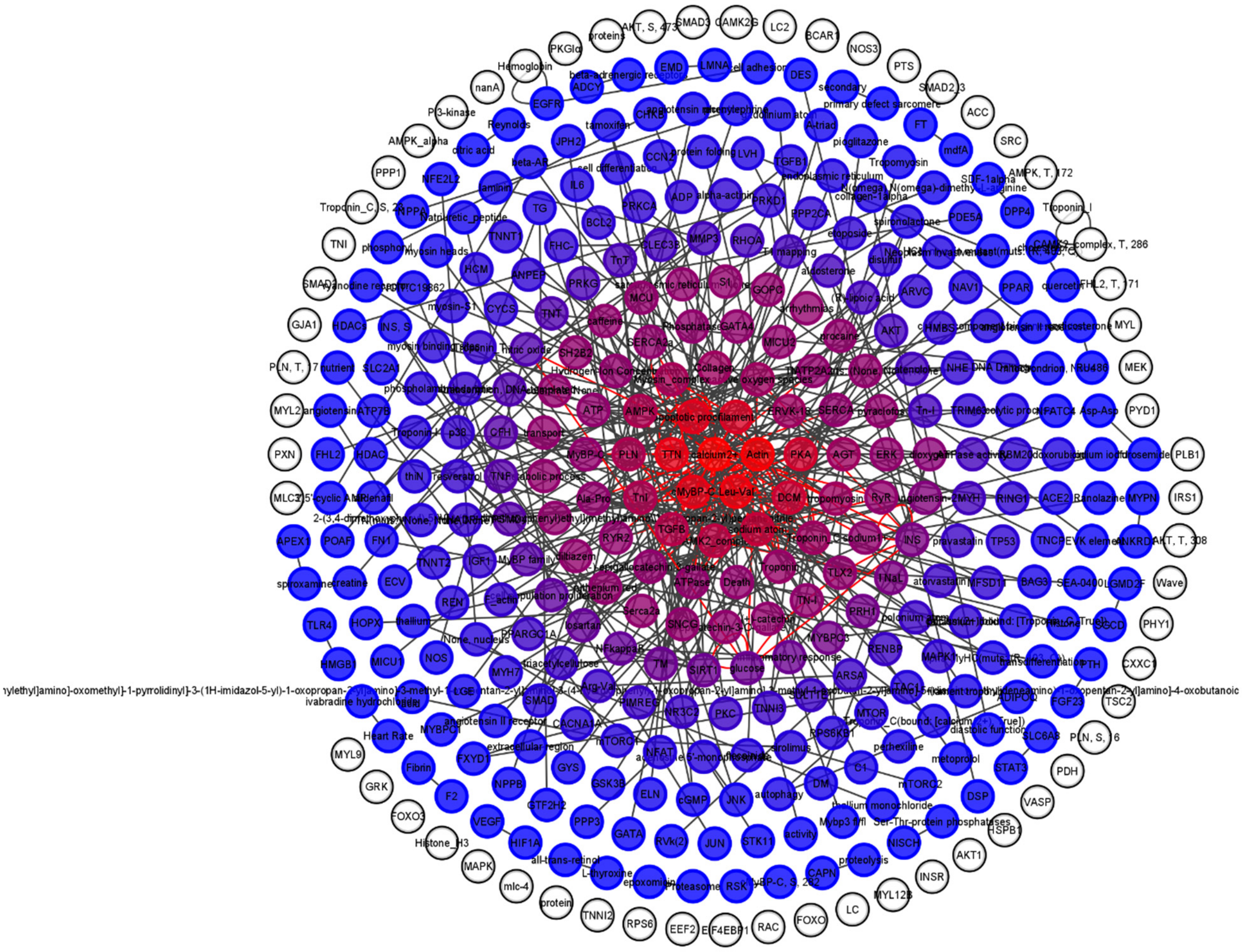

Hypertrophic cardiomyopathy ∩ atrial fibrillation

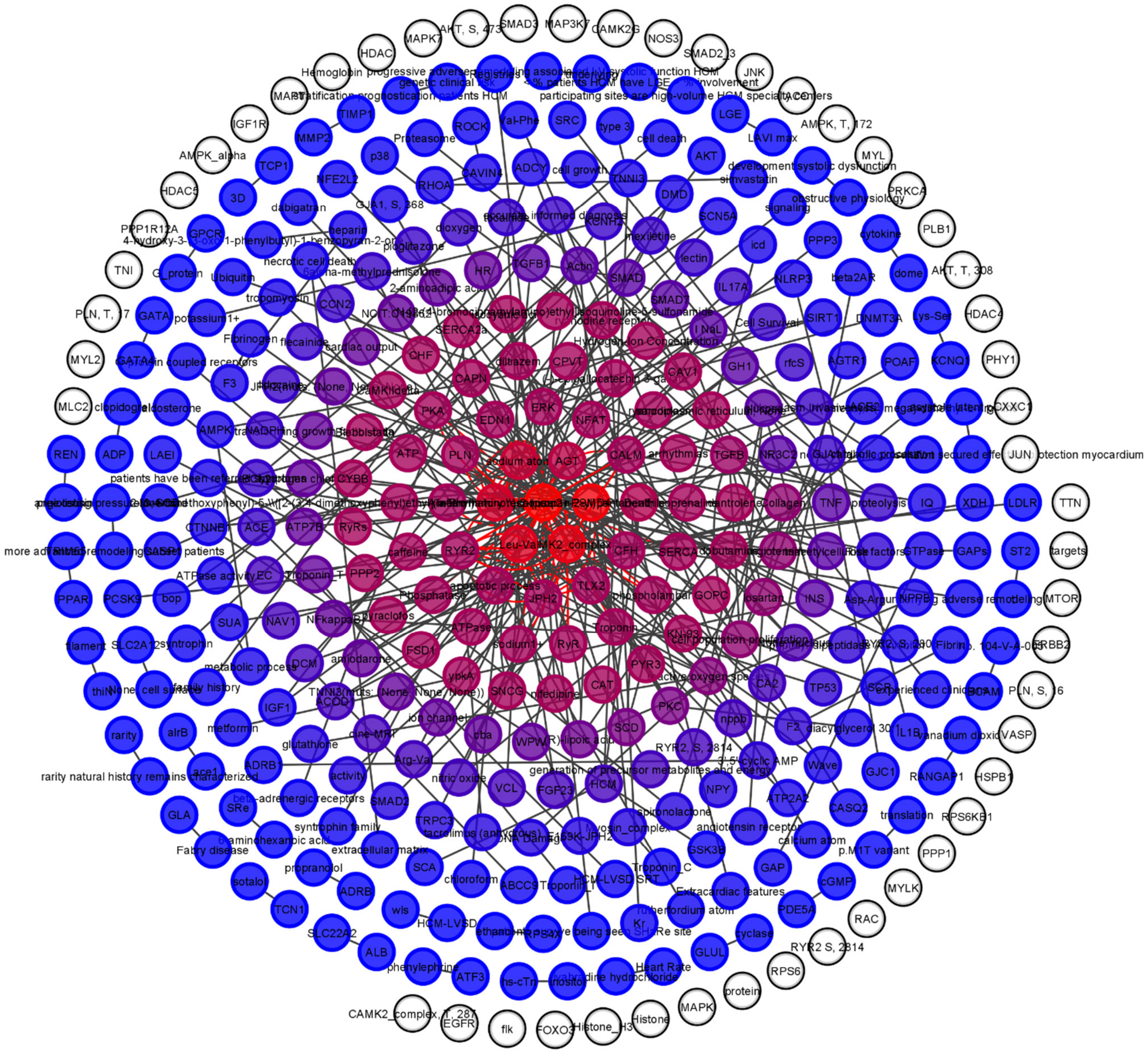

Hypertrophic cardiomyopathy ∩ sudden cardiac death

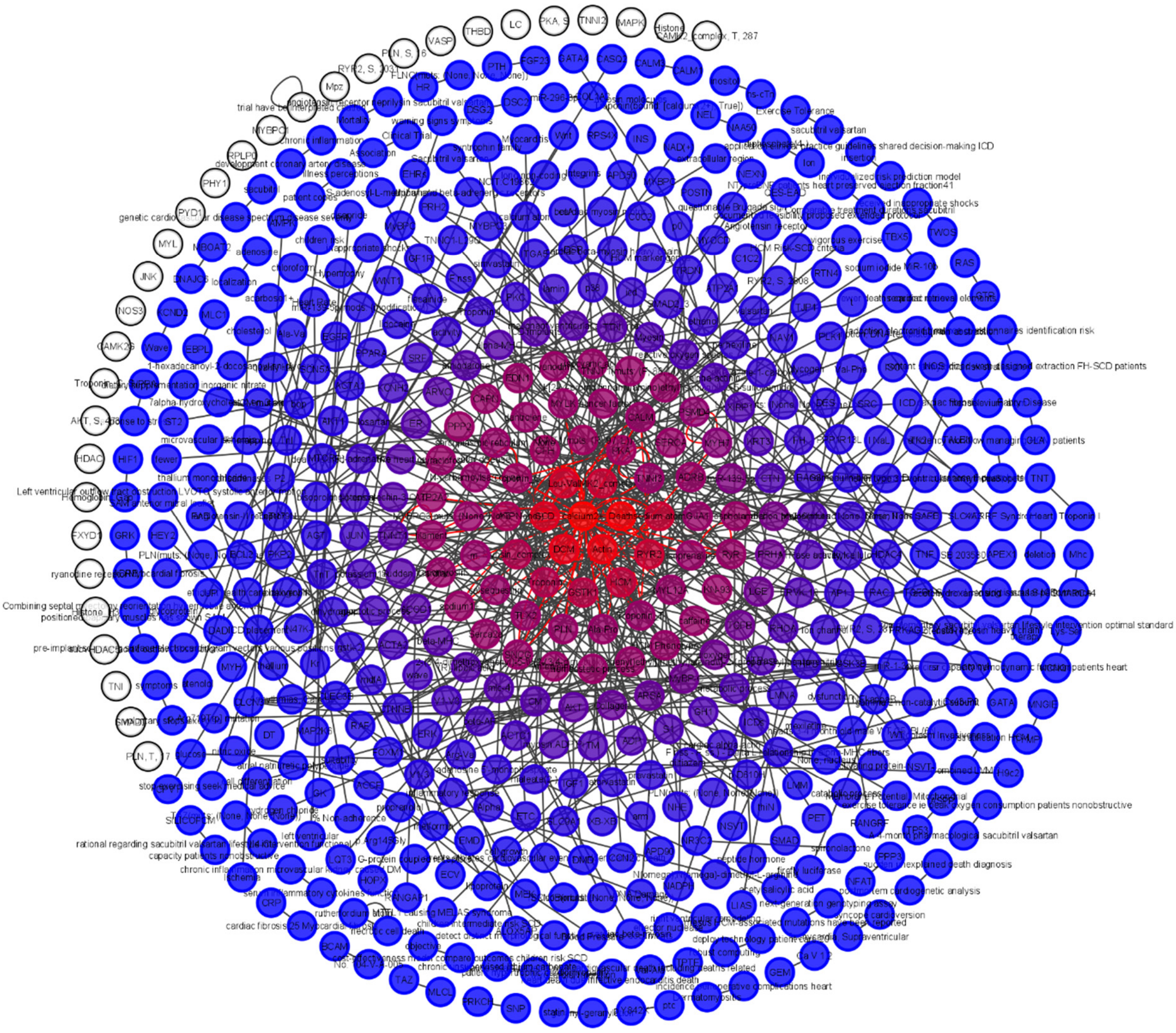

Hypertrophic cardiomyopathy ∩ coronary microvascular dysfunction

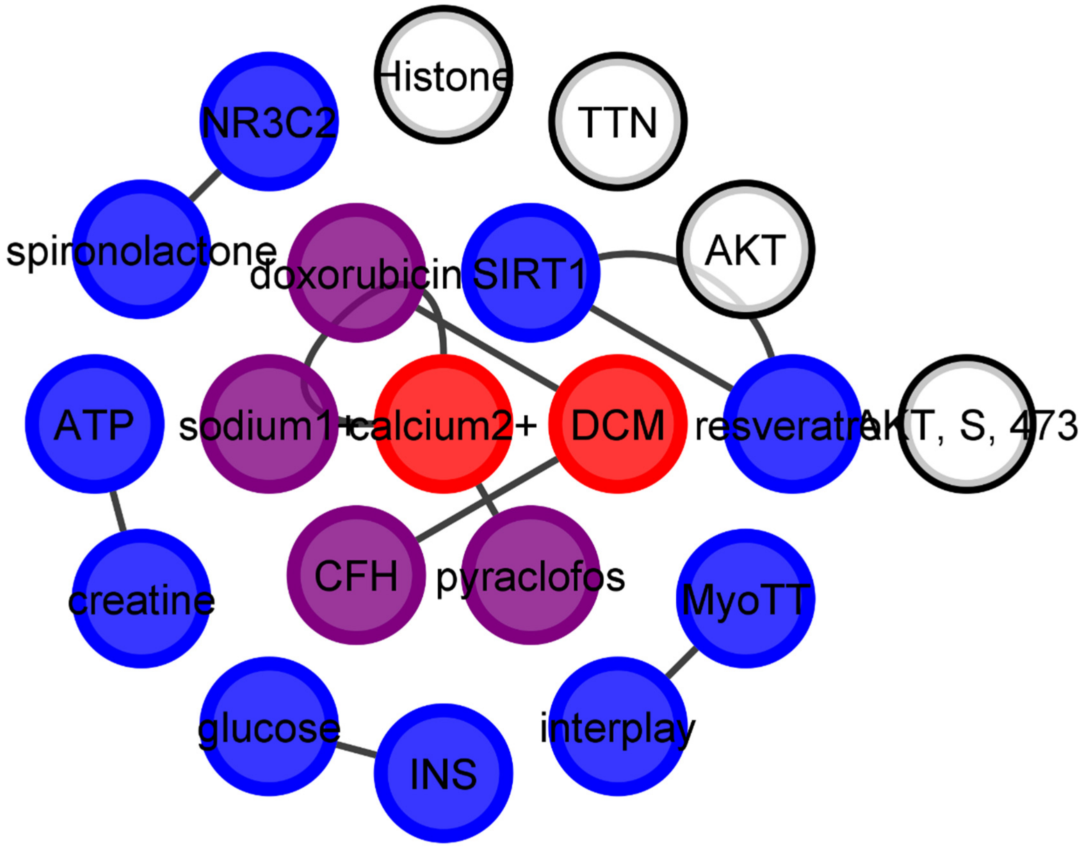

Hypertrophic cardiomyopathy ∩ myocardial ischemia

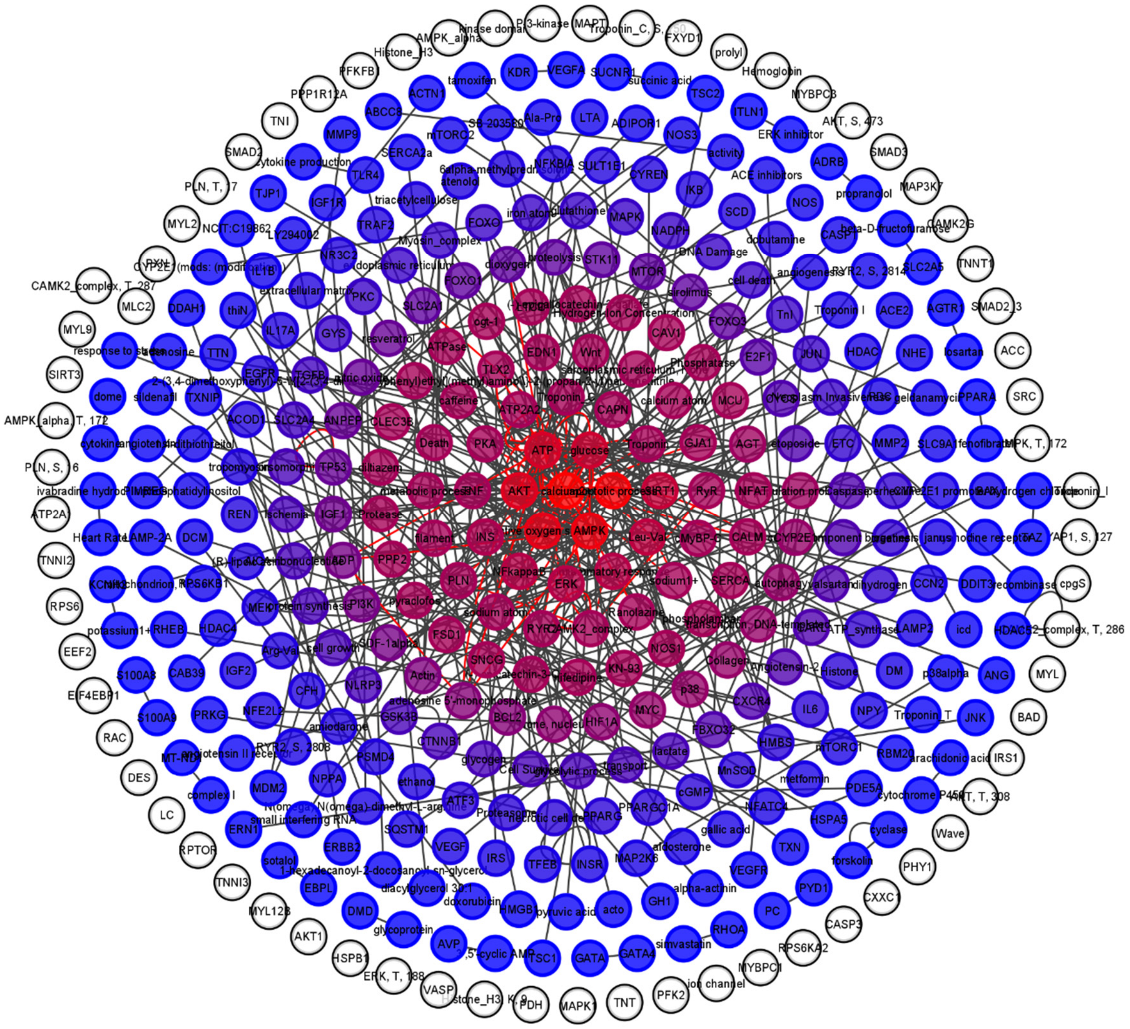

Hypertrophic cardiomyopathy ∩ heart failure

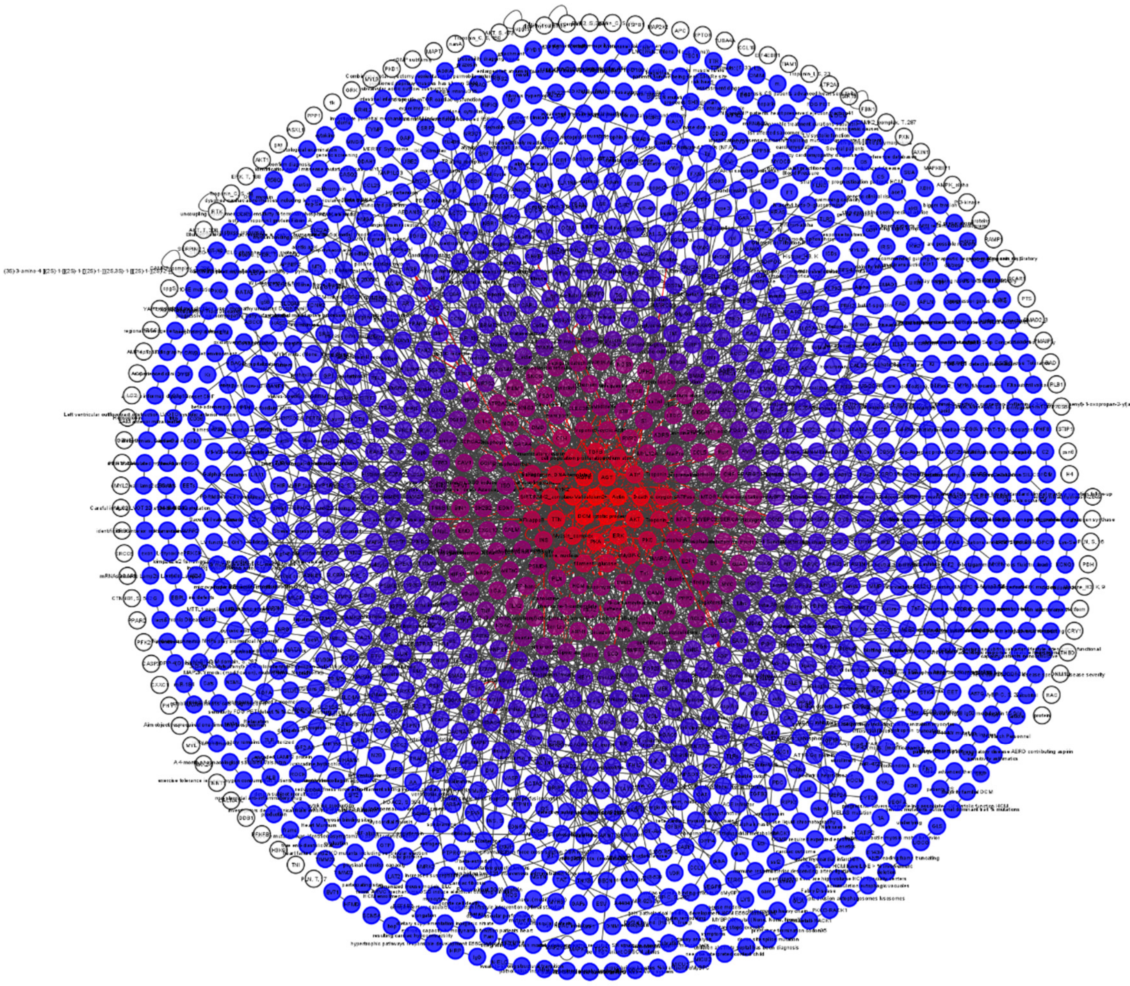

Hypertrophic cardiomyopathy ∩ major adverse cardiovascular events

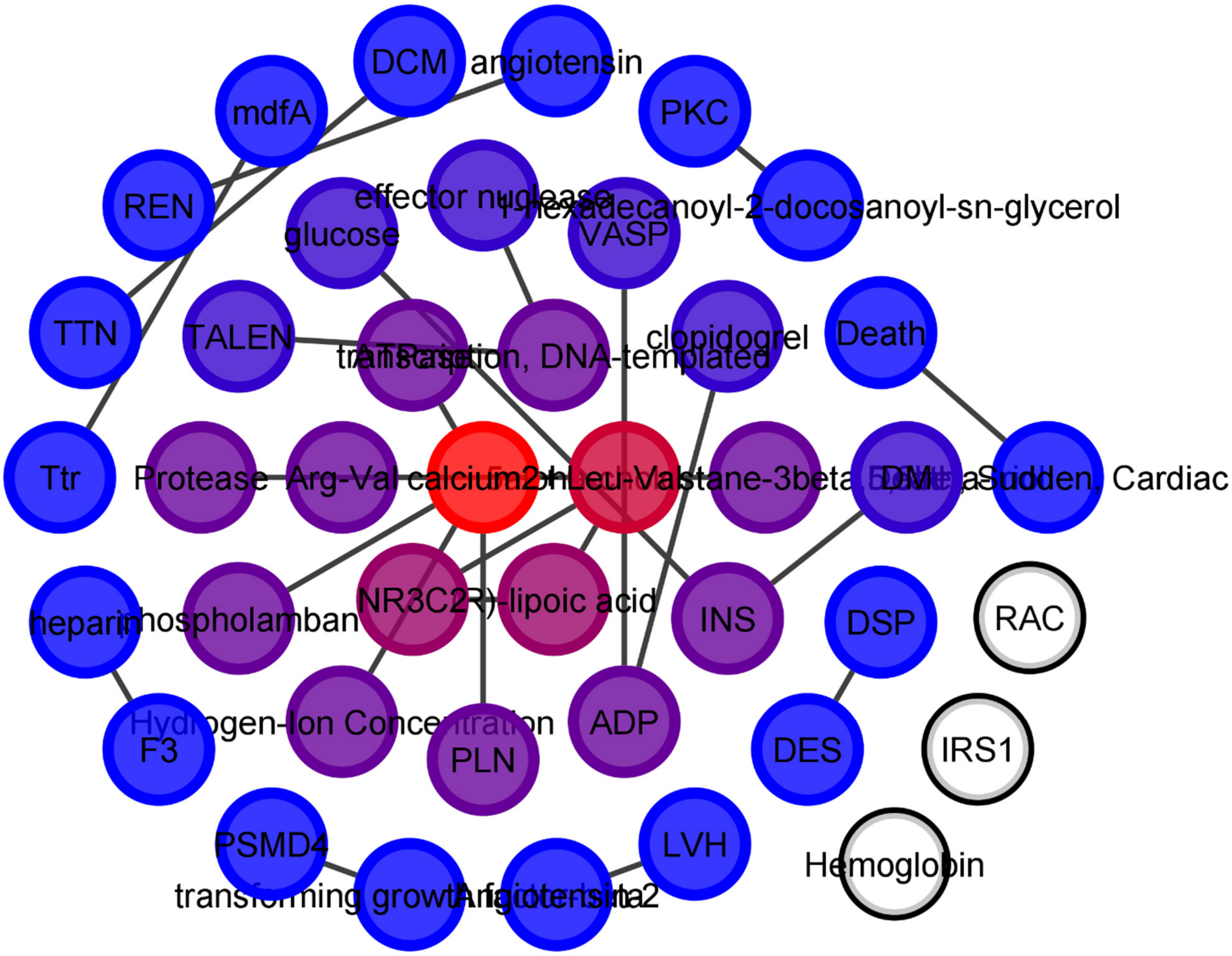

Hypertrophic cardiomyopathy ∩ rehospitalization

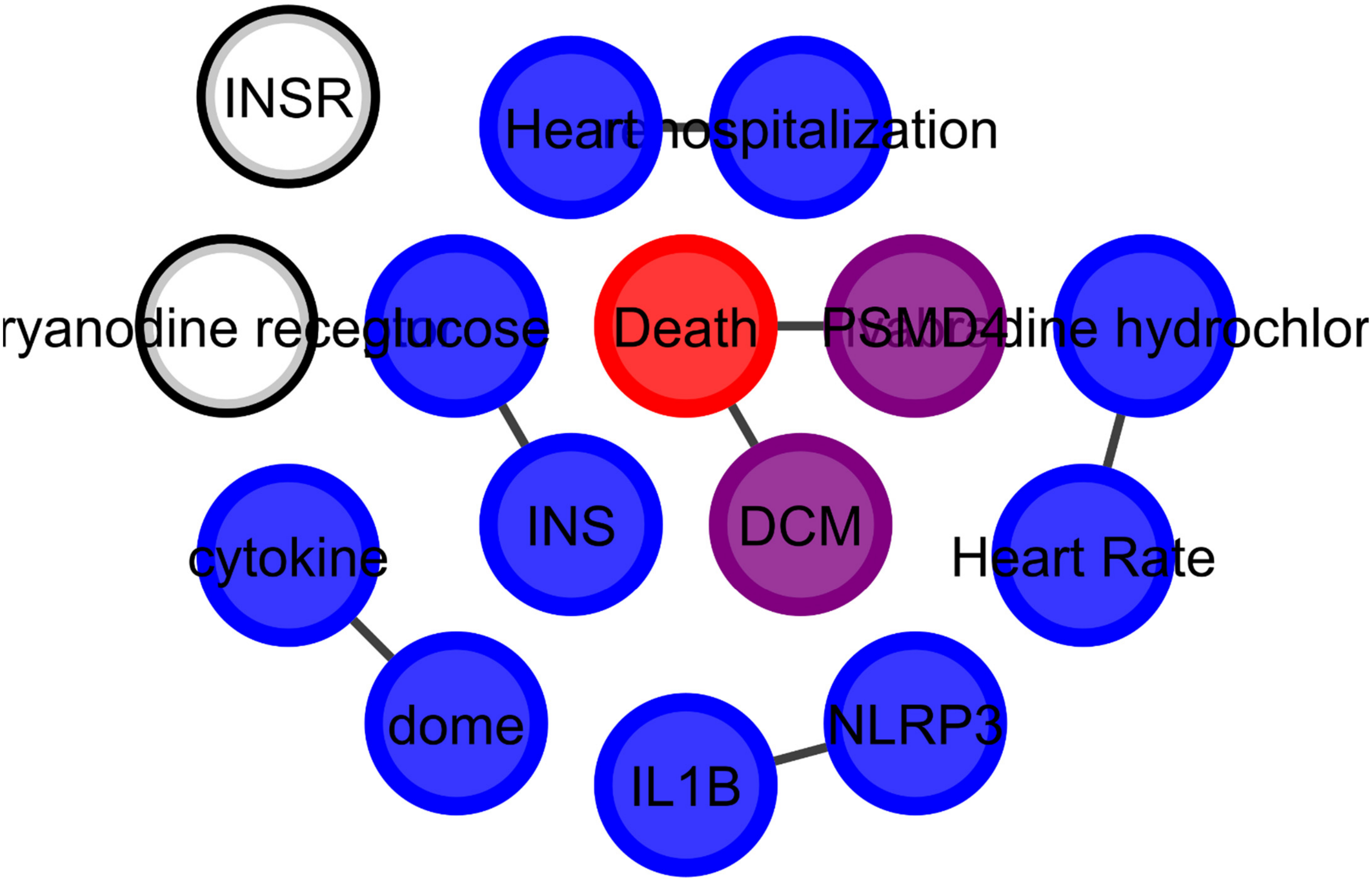

Figure S1. The most important nodes in networks represented as packed concentric rings sorted by the most important nodes. K-shell decomposition method ranks the most important nodes in a network and partitions them into shells based on that rank. Networks are presented as packed concentric rings sorted by k-shell. Gradient to the color of nodes is applied by k-shell: the closer to the center node is, and the redder node is—the more important it is.
