# Supplementary material for: Shared Molecular Mechanisms of Hypertrophic Cardiomyopathy and Its Clinical Presentations: Automated Molecular Mechanisms Extraction Approach
Source: Life (Basel). 2021 Aug 3;11(8):785. doi: 10.3390/life11080785 (PMC8398249; doi:10.3390/life11080785)
Supplement: Supplementary file 1 [file life-11-00785-s001.zip › Table S2.pdf]

Table S2. Cooperatively working elements (functional modules).

hypertrophic cardiomyopathy ∩ cardiomyocyte hypertrophy Page rank	Members	Cliqueness	# of children
	ATP2A2, PKA, PLN, calcium2+	0.833	0
	AGT, Collagen, TGFB, cell population proliferation	0.833	0

hypertrophic cardiomyopathy ∩ cardiomyocyte hypertrophy Node degree	Members	Cliqueness	# of children
	ATP2A2, PKA, PLN, calcium2+	0.833	0
	AGT, Collagen, TGFB, cell population proliferation	0.833	0

hypertrophic cardiomyopathy ∩ myofibrillar disarray Page rank	Members	Cliqueness	# of children
	Actin, MYL12A, Myosin_complex	1.000	0
	Ad, DUSP6, ERK	1.000	0
	NFAT, PPP3, cyclosporin A	1.000	0

hypertrophic cardiomyopathy ∩ myofibrillar disarray Node degree	Members	Cliqueness	# of children
	Actin, MYL12A, Myosin_complex	1.000	0
	NFAT, PPP3, cyclosporin A	1.000	0

hypertrophic cardiomyopathy ∩ cardiomyocyte disarray, page rank – none

hypertrophic cardiomyopathy ∩ cardiomyocyte disarray, node degree – none

hypertrophic cardiomyopathy ∩ myocardial remodeling Page rank	Members	Cliqueness	# of children
	CALM, CAMK2_complex, calcium2+, sodium atom	0.833	0
	AGT, Collagen, TGFB, cell population proliferation	0.833	0
	AGT, apoptotic process, inflammatory response	1.000	0

hypertrophic cardiomyopathy ∩ myocardial remodeling Node degree	Members	Cliqueness	# of children
	CALM, CAMK2_complex, calcium2+, sodium atom	0.833	0
	AGT, Collagen, TGFB, cell population proliferation	0.833	0
	AGT, apoptotic process, inflammatory response	1.000	0

hypertrophic cardiomyopathy ∩ cardiac remodeling Page rank	Members	Cliqueness	# of children
	TNF, apoptotic process, p38, transcription, DNA-templated	0.833	0
	AMPK, SIRT1, glucose, resveratrol	0.833	0
	AGT, CCN2, Collagen, TGFB, cell population proliferation	0.800	0
	AGT, apoptotic process, inflammatory response	1.000	0
	AKT, IGF-1, apoptotic process	1.000	0
	AKT, AMPK, MTOR	1.000	0
	NADPH, dioxygen, reactive oxygen species	1.000	0
	CAMK2_complex, PKA, PLN, SERCA, calcium2+	0.700	0
	PKA, PLN, Troponin_C, calcium2+	0.833	0

hypertrophic cardiomyopathy ∩ cardiac remodeling Node degree	Members	Cliqueness	# of children

	AKT, AMPK, MTOR	1.000	0
	AGT, apoptotic process, inflammatory response	1.000	0
	AKT, IGF-1, apoptotic process	1.000	0
	AGT, CCN2, Collagen, TGFB, cell population proliferation	0.800	0
	AMPK, SIRT1, glucose	1.000	0
	CALM, CAMK2_complex, PLN, RYR2, RyR, calcium2+	0.667	0
	TNF, apoptotic process, p38, transcription, DNA-templated	0.833	0
	CAMK2_complex, PKA, PLN, SERCA, calcium2+	0.700	0
	PKA, PLN, Troponin_C, calcium2+	0.833	0

hypertrophic cardiomyopathy ∩ myocardial fibrosis Page rank	Members	Cliqueness	# of children
	AGT, apoptotic process, inflammatory response	1.000	0
	CAMK2_complex, TLX2, calcium2+, sodium atom	0.833	0
	AGT, Angiotensin-2, CCN, Collagen, TGFB, cell population proliferation	0.733	0
	Apoptotic process, p38, transcription, DNA-templated	1.000	0
	AMPK, INS, glucose	1.000	0
	ATPase, Actin, Myosin_complex	1.000	0
	AKT, IGF1, apoptotic process	1.000	0

hypertrophic cardiomyopathy ∩ myocardial fibrosis Node degree	Members	Cliqueness	# of children
	CAMK2_complex, TLX2, calcium2+, sodium atom	0.833	0
	AGT, apoptotic process, inflammatory response	1.000	0
	AGT, Angiotensin-2, CCN2, Collagen, TGFB, cell population proliferation	0.733	0
	AMPK, INS, glucose	1.000	0
	AKT, IGF1, apoptotic process	1.000	0
	Apoptotic process, p38, transcription, DNA-templated	1.000	0
	SMAD, SMAD7, TGFB	1.000	0
	ATPase, Actin, Myosin_complex	1.000	0

hypertrophic cardiomyopathy ∩ left ventricular outflow tract obstruction, page rank – none

hypertrophic cardiomyopathy ∩ left ventricular outflow tract obstruction, node degree – none

hypertrophic cardiomyopathy ∩ impaired myocardial relaxation, page rank. – none

hypertrophic cardiomyopathy ∩ impaired myocardial relaxation, node degree – none

hypertrophic cardiomyopathy ∩ myocardial stiffness, page rank – none

hypertrophic cardiomyopathy ∩ myocardial stiffness, node degree – none

hypertrophic cardiomyopathy ∩ diastolic dysfunction Page rank	Members	Cliqueness	# of children
	ATPase, Actin, TnI, Troponin, cMyBP-C, calcium2+, filament	0.619	0
*	Actin, Myosin_complex, Troponin, cMyBP-C, calcium2+, filament	0.800	2
Child of *	Actin, Myosin_complex, Troponin, cMyBP-C, calcium2+, filament, thiN	0.619	0
Child of *	Actin, ERVK-18, Myosin_complex, Troponin, cMyBP-C, calcium2+, filament, tropomyosin	0.607	0
	Ala-Pro, CAMK2_complex, I NaL, TLX2, calcium2+, sodium atom	0.667	0
	Actin, TNT, Troponin, cMyBP-C, tropomyosin	0.700	0
	Ala-Pro, I NaL, calcium2+, sodium atom, sodium1+	0.700	0
	Actin, Myosin_complex, PRH1, cMyBP-C, calcium2+, filament, tropomyosin	0.619	0
	Actin, ERVK-18, Myosin_complex, cMyBP-C, filament	0.800	0
	(R)-lipoic acid, Leu-Val, PSMD4, atenolol	0.833	0
	(-)-epicatechin-3-O-gallate, Death, calcium2+	1.000	0
	Actin, MyBP-C, PKA, TnI, Calcium2+	0.800	0
	AGT, Angiotensin-2, Collagen, TGFB, cell population proliferation	0.700	0
	ATP2A2, CAMK2_complex, PLN, RYR2, SERCA2a, calcium2+	0.600	0
	PLN, RYR2, calcium2+	1.000	0
	PKA, TNNI3, Troponin_C, calcium2+	0.833	0
	INS, glucose, nitric oxide, reactive oxygen species	0.833	0
	(-)-epigallocatechin 3-gallate, ATPase, Actin, calcium2+	0.833	0
	Actin, Troponin, calcium2+, filament, tropomyosin	0.800	0

	TM, TnT, Troponin_C	1.000	0
	CLEC3B, DCM, ERVK-18, FHC-	0.833	0
	Angiotensin-2, TGFB, cell population proliferation	1.000	0
	CAMK2_complex, I NaL, PLN, RyR, calcium2+, sodium atom	0.600	0
	NFkappaB, SIRT1, TNF, apoptotic process	0.833	0
	CLEC3B, DCM, ERVK-18, FHC-	0.833	0
	Angiotensin-2, TGFB, cell population proliferation	1.000	0
	CAMK2_complex, I NaL, PLN, RyR, calcium2+, sodium atom	0.600	0
	NFkappaB, SIRT1, TNF, apoptotic process	0.833	0
	TLX2, calcium2+, sodium atom	1.000	0
	PRH1, cMyBP-C, filament	1.000	0
	PKA, PLN, TnI, Troponin_C, cMyBP-C, calcium2+	0.600	0

hypertrophic cardiomyopathy ∩ diastolic dysfunction Node degree	Members	Cliqueness	# of children
*	PKA, TnI, Troponin_C, cMyBP-C, calcium2+	0.700	2
Child of *	Actin, PKA, TnI, Troponin_C, cMyBP-C, calcium2+, filament	0.619	0
Child of *	PKA, PLN, TnI, Troponin_C, cMyBP-C, calcium2+	0.600	0
	Actin, Myosin_complex, PRH1, Troponin, cMyBP-C, calcium2+, filament, tropomyosin	0.607	0
	(R)-lipoic acid, Leu-Val, PSMD4, atenolol	0.833	0
	Ala-Pro, CAMK2_complex, I NaL, TLX2, calcium2+, sodium atom	0.667	0
	CAMK2_complex, I NaL, PLN, RyR, calcium2+, sodium atom	0.600	0
	AGT, Angiotensin-2, Collagen, TGFB, cell population proliferation	0.700	0
	PKA, TNNI3, Troponin_C, calcium2+	0.833	0
	Actin, ERVK-18, Myosin_complex, cMyBP-C, filament	0.800	0
	Actin, MyBP-C, PKA, TnI, calcium2+	0.800	0
	Actin, TNT, Troponin, cMyBP-C, tropomyosin	0.700	0
	INS, glucose, nitric oxide, reactive oxygen species	0.833	0
	ATP2A2, CAMK2_complex, PKA, PLN, RYR2, calcium2+	0.600	0
	Ala-Pro, I NaL, calcium2+, sodium atom, sodium1+	0.700	0
	NFkappaB, SIRT1, TNF, apoptotic process	0.833	0
	PLN, RYR2, calcium2+	1.000	0
	(-)-epigallocatechin 3-gallate, ATPase, Actin, calcium2+	0.833	0
	(-)-epicatechin-3-O-gallate, Death, calcium2+	1.000	0

	PRH1, cMyBP-C, filament	1.000	0
	TM, TnT, Troponin_C	1.000	0
	CLEC3B, DCM, ERVK-18, FHC-	0.833	0
	TLX2, calcium2+, sodium atom	1.000	0

hypertrophic cardiomyopathy ∩ atrial fibrillation Page rank	Members	Cliqueness	# of children
*	Ala-Pro, CFH, Death, PSMD4, calcium2+	0.700	2
Child of *	Ala-Pro, CFH, Death, PSMD4, calcium2+, sodium atom	0.667	0
Child of *	Ala-Pro, CFH, Death, PSMD4, SCD, calcium2+	0.600	0
	Ala-Pro, CAMK2_complex, CFH, calcium2+, sodium atom, sodium1+	0.600	0
	(R)-lipoic acid, Leu-Val, NR3C2, PSMD4, SCD	0.700	0
	Death, PSMD4, SCD, amiodarone, calcium2+	0.700	0
	Ala-Pro, CFH, PSMD4, calcium2+, sodium atom	0.800	0
	CALM, CAMK2_complex, JPH2, RYR2, TLX2, calcium2+	0.733	0
	CALM, CAMK2_complex, PLN, RYR2, calcium2+, sodium atom	0.667	0
	Death, Leu-Val, PSMD4, SCD	0.833	0
	JPH2, PSMD4, RYR2, TLX2, calcium2+	0.800	0
	PKA, PLN, PSMD4, Troponin, calcium2+	0.700	0
	Angiotensin-2, Collagen, TGFB, cell population proliferation, losartan	0.800	0
	CAMK2_complex, PKA, PLN, SERCA, calcium2+	0.700	0
	AGT, apoptotic process, inflammatory response	1.000	0
	SMAD, SMAD7, TGFB	1.000	0
	NFkappaB, TNF, apoptotic process, inflammatory response	0.833	0

hypertrophic cardiomyopathy ∩ atrial fibrillation Node degree	Members	Cliqueness	# of children
	CAMK2_complex, Death, JPH2, PSMD4, RYR2, calcium2+	0.600	2
	CFH, Death, JPH2, PSMD4, SCD, calcium2+	0.600	0
	CALM, CAMK2_complex, PLN, RYR2, calcium2+, sodium atom	0.667	0
	(R)-lipoic acid, Leu-Val, NR3C2, PSMD4, SCD	0.700	0
	Ala-Pro, CAMK2_complex, CFH, Phosphatase, calcium2+, sodium atom	0.600	0
	AGT, apoptotic process, inflammatory response	1.000	0

	Death, PSMD4, SCD, amiodarone, calcium2+	0.700	0
	Angiotensin-2, Collagen, TGFB, cell population proliferation, losartan	0.800	0
	CALM, CAMK2_complex, JPH2, RYR2, TLX2, calcium2+	0.733	0
	JPH2, PSMD4, RYR2, TLX2, calcium2+	0.800	0
	CAMK2_complex, PKA, PLN, SERCA, calcium2+	0.700	0
	Ala-Pro, CFH, PSMD4, calcium2+, sodium atom	0.800	0
	PKA, PLN, PSMD4, Troponin, calcium2+	0.700	0
	Death, Leu-Val, PSMD4, SCD	0.833	0
	NFkappaB, TNF, apoptotic process, inflammatory response	0.833	0
	SMAD, SMAD7, TGFB	1.000	0

hypertrophic cardiomyopathy ∩ sudden cardiac death Page rank	Members	Cliqueness	# of children
	Death, PKA, TNNI3, Troponin_C, Troponin_T, calcium2+	0.600	0
	Death, Troponin_T, calcium2+	1.000	0
	Ala-Pro, calcium2+, isoprenaline, potassium1+, sodium atom, sodium1+	0.600	0
	Ala-Pro, CAMK2_complex, TLX2, calcium2+, sodium atom	0.700	0
	CAMK2_complex, CFH, DCM, RYR2, SNCG, calcium2+	0.600	0
	ARSA, CM, Death, Leu-Val, PSMD4, SCD	0.667	0
	CFH, Death, Leu-Val, PSMD4, SCD	0.800	0
	CALM, MYL12A, MYLK, calcium2+	0.833	0
	Actin, MYL12A, Myosin_complex, Troponin, tropomyosin	0.700	0
	CALM, CAMK2_complex, PLN, RYR2, calcium2+, sodium atom	0.667	0
	THPO, TNNT1(muts:(R,97,L)), alpha-MHC, beta-MHC, phenazine-1-carboxylate	0.700	0
	TNNI3, Troponin_T, calcium2+	1.000	0
	RYR2, SNCG, Troponin, calcium2+	0.833	0
	Actin, Myosin_complex, SNCG, Troponin, calcium2+, tropomyosin	0.600	0
	Ala-Pro, potassium1+, sodium1+	1.000	0
	ATP2A2, CAMK2_complex, PKA, PLN, calcium2+	0.700	0
	CALM, CAMK2_complex, MYLK, RYR2, calcium2+	0.800	0
	AKT1, MTOR, SMAD2_3	1.000	0
	ARSA, Death, SCD	1.000	0
	JUN, isoprenaline, miR-139-5p	1.000	0

	GSTK1, HCM, MYH7, SRF	0.833	0
	Ala-Pro, calcium2+, isoprenaline	1.000	0
	V1-3, V1-V3, wave	1.000	0
	PKA, PLN, Troponin_C, calcium2+	0.833	0

hypertrophic cardiomyopathy ∩ sudden cardiac death Node degree	Members	Cliqueness	# of children
	Death, PKA, TNNI3, Troponin_C, Troponin_T, calcium2+	0.600	0
	Death, Troponin_T, calcium2+	1.000	0
	Actin, MYL12A, Myosin_complex, Troponin, tropomyosin	0.700	0
	ARSA, CM, Death, Leu-Val, PSMD4, SCD	0.667	0
	CFH, Death, Leu-Val, PSMD4, SCD	0.800	0
	GSTK1, HCM, MYH7, SRF	0.833	0
	CALM, CAMK2_complex, PLN, RYR2, calcium2+, sodium atom	0.667	0
	Ala-Pro, CAMK2_complex, TLX2, calcium2+, sodium atom	0.700	0
	CALM, CAMK2_complex, CFH, DCM, RYR2, calcium2+	0.667	0
	Actin, Myosin_complex, SNCG, Troponin, calcium2+, tropomyosin	0.600	0
	THPO, TNNT1(muts:(R,97,L)), alpha-MHC, beta-MHC, phenazine-1-carboxylate	0.700	0
	Ala-Pro, calcium2+, isoprenaline	1.000	0
	PKA, PLN, Troponin_C, calcium2+	0.833	0
	TNNI3, Troponin_T, calcium2+	1.000	0
	JUN, isoprenaline, miR-139-5p	1.000	0
	Ala-Pro, calcium2+, isoprenaline, potassium1+, sodium atom, sodium1+	0.600	0
	ATP2A2, CAMK2_complex, PKA, PLN, calcium2+	0.700	0
	CALM, MYL12A, MYLK, calcium2+	0.833	0
	CALM, CAMK2_complex, MYLK, RYR2, calcium2+	0.800	0
	V1-3, V1-V3, wave	1.000	0
	RYR2, SNCG, Troponin, calcium2+	0.833	0
	ARSA, Death, SCD	1.000	0
	Ala-Pro, potassium1+, sodium1+	1.000	0
	AKT1, MTOR, SMAD2_3	1.000	0

hypertrophic cardiomyopathy ∩ coronary microvascular dysfunction, page rank – none

hypertrophic cardiomyopathy ∩ coronary microvascular dysfunction, node degree – none

hypertrophic cardiomyopathy ∩ myocardial ischemia Page rank	Members	Cliqueness	# of children
	CAPN, apoptotic process, calcium2+	1.000	0
	AMPK, ATP, INS, adenosine 5′-monophosphate, glucose, metabolic process	0.600	0
	CYP2E1, apoptotic process, reactive oxygen species	1.000	0
	AMPK, ATP, INS, SLC2A1, glucose, reactive oxygen species	0.600	0
	AKT, CYP2E1, ERK, MYC, PI3K	0.700	0
	NFkappaB, SIRT1, TNF, apoptotic process, inflammatory response	0.700	0
	AMPK, INS, glucose	1.000	0
	Ranolazine, TLX2, calcium2+, sodium atom, sodium1+	0.700	0
	EDN1, ERK, calcium2+	1.000	0
	NFkappaB, TNF, apoptotic process, p38	0.833	0
	HIF1A, lactate, metabolic process	1.000	0
	CALM, CAMK2_complex, RYR2, calcium2+	0.833	0
	ADP, AMPK, ATP, STK11, adenosine 5′-monophosphate, metabolic process	0.667	0
	PKA, PLN, Troponin, Troponin_C, calcium2+	0.700	0
	CAMK2_complex, PLN, RYR2, RyR, calcium2+	0.700	0
	AKT, IGF1, apoptotic process	1.000	0
	ATP, dioxygen, reactive oxygen species	1.000	0
	ATP2A2, CAMK2_complex, PKA, PLN, calcium2+	0.700	0
	CXCR4, SDF-1alpha, cell population proliferation	1.000	0
	Actin, Myosin_complex, filament	1.000	0
	AMPK, STK11, adenosine 5′-monophosphate	1.000	0

hypertrophic cardiomyopathy ∩ myocardial ischemia Node degree	Members	Cliqueness	# of children
	CAPN, apoptotic process, calcium2+	1.000	0
	AKT, IGF1, apoptotic process	1.000	0
	CYP2E1, apoptotic process, reactive oxygen species	1.000	0
	AMPK, ATP, INS, SLC2A1, glucose, reactive oxygen species	0.600	0

	NFkappaB, SIRT1, TNF, apoptotic process, inflammatory response	0.700	0
	EDN1, ERK, calcium2+	1.000	0
	NFkappaB, apoptotic process, inflammatory response	1.000	0
	NFkappaB, TNF, apoptotic process, p38	0.833	0
	AMPK, INS, glucose	1.000	0
	PKA, PLN, Troponin, Troponin_C, calcium2+	0.700	0
	Actin, Myosin_complex, filament	1.000	0
	CAMK2_complex, PLN, RYR2, RyR, calcium2+	0.700	0
	AKT, CYP2E1, ERK, MYC, P13K	0.700	0
	ATP2A2, CAMK2_complex, PKA, PLN, calcium2+	0.700	0
	Ranolazine, TLX2, calcium2+, sodium atom, sodium1+	0.700	0
	ADP, AMPK, ATP, STK11, adenosine 5′-monophosphate, metabolic process	0.667	0
	AKT, ERK, SDF-1alpha	1.000	0
	CALM, CAMK2_complex, RYR2, calcium2+	0.833	0
	HIF1A, lactate, metabolic process	1.000	0
	CXCR4, SDF-1alpha, cell population proliferation	1.000	0
	ATP, dioxygen, reactive oxygen species	1.000	0
	AMPK, STK11, adenosine 5′-monophosphate	1.000	0

hypertrophic cardiomyopathy ∩ heart failure Page rank	Members	Cliqueness	# of children
	CAPN, apoptotic process, calcium2+	1.000	0
	NFkappaB, TNF, apoptotic process, p38, transcription, DNA-templated	0.700	0
	DCM, Death, HCM, Leu-Val, PSMD4, SCD, calcium2+	0.619	0
	CFH, DCM, Death, Leu-Val, Myosin_complex, cMyBP-C, calcium2+	0.619	0
	Ala-Pro, CAMK2_complex, CFH, I NaL, PKC, calcium2+, sodium atom, sodium1+	0.607	0
*	(R)-lipoic acid, CFH, Death, Leu-Val, PSMD4	0.900	2
Child of *	(R)-lipoic acid, CFH, Death, Leu-Val, PSMD4, SCD	0.800	0
Child of *	(R)-lipoic acid, CFH, Death, LIAS, Leu-Val, NR3C2, PSMD4	0.619	0
	CFH, DCM, Leu-Val, PSMD4, calcium2+, sodium atom	0.600	0
	Actin, MYL12A, Myosin_complex, Troponin, cMyBP-C, calcium2+, filament, thiN	0.643	0
	ATP, Actin, NADH, S1, calcium2+	0.700	0
	Ala-Pro, I NaL, Troponin_T, calcium2+, sodium atom, sodium1+	0.667	0

*	ATPase, Actin, Myosin_complex, TTN, Troponin_C, cMyBP-C, filament	0.619	2
Child of *	ATPase, Actin, DCM, Myosin_complex, TTN, Troponin_C, cMyBP-C, filament	0.607	0
Child of *	ATPase, Actin, Myosin_complex, TTN, Troponin, Troponin_C, cMyBP-C, calcium2+, filament, tropomyosin	0.600	0
	EDN1, ERK, NPPA, PKC, PLN, calcium2+	0.600	0
	AGT, Angiotensin-2, CCN2, Collagen, POSTN, TGFB, cell population proliferation, losartan	0.607	0
	Actin, DCM, Leu-Val, Myosin_complex, PRH1, cMyBP-C, calcium2+, filament	0.607	0
	CAMK2_complex, CFH, I NaL, RYR2, calcium2+, sodium atom, transcription, DNA-templated	0.619	0
	Ala-Pro, DCM, TnI, Troponin_C, Troponin_T, calcium2+, tropomyosin	0.619	0
	AGT, NFkappaB, apoptotic process, dioxygen, inflammatory response, triacetylcellulose	0.600	0
	Actin, DCM, MYBPC3, Myosin_complex, PKA, TTN	0.667	0
	ATPase, Actin, ERVK-18, MYL12A, Myosin_complex, TNNI3, Troponin_C, calcium2+	0.607	0
	ATP, INS, apoptotic process, dioxygen, glucose, reactive oxygen species	0.600	0
	ERK, PKC, TnI, cMyBP-C, calcium2+, sodium atom	0.600	0
	AGT, AMPK, INS, apoptotic process, glucose, reactive oxygen species	0.600	0
	Actin, Myosin_complex, PSMD4, Troponin, Troponin_C, calcium2+, filament, tropomyosin	0.643	0
	AKT, IGF1, apoptotic process	1.000	0
	AMPK, ATP, INS, cell population proliferation, glucose, reactive oxygen species	0.667	0
	TNF, cell death, necrotic cell death	1.000	0
	Actin, Myosin_complex, TNT, Troponin, Troponin_T, calcium2+, tropomyosin	0.619	0
	DCM, Death, HCM, MYH7, TNNI3, calcium2+	0.667	0
	NFkappaB, SIRT1, SIRT6, TNF, apoptotic process, inflammatory response	0.600	0
	EDN1, GATA4, NFAT, Phosphatase, calcium2+	0.700	0
	ATPase, Actin, DCM, Myosin_complex, TNNI3, TnI, Troponin, Troponin_C, Troponin_T, calcium2+	0.600	0
	Actin, PKA, PKC, TnI, Troponin_C, Troponin_T, calcium2+	0.667	0
	CAMK2_complex, CFH, DCM, JPH2, RYR2, SNCG, calcium2+	0.619	0
	Ala-Pro, Ranolazine, calcium2+, potassium1+, sodium atom, sodium1+	0.667	0
	ADRB, GRK2, Gly-Gln, PKC, calcium2+	0.700	0

	ATPase, MYL12A, Myosin_complex, PKC, calcium2+, filament	0.667	0
	BMP10, SMARCA4, cell population proliferation	1.000	0
	ERK, MEK, RAF, RAF1, microtubule	0.700	0
	Angiotensin-2, Collagen, TGFB, cell population proliferation	0.833	0
	(R)-lipoic acid, LIAS, Leu-Val	1.000	0
	TFEB, localization, mTORC1	1.000	0
	BIN1, CAV1, calcium2+	1.000	0
	ATP, NADPH, dioxygen, reactive oxygen species	0.833	0
	DSC2, DSG2, desmosomal proteins	1.000	0
	TLX2, calcium2+, sodium atom	1.000	0
	EDN1, ERK, NFAT, NPPA, calcium2+	0.700	0
	AKT, AMPK, MTOR	1.000	0
	PRKAG2, adenosine 5′-monophosphate, glycogen	1.000	0
	DPF3a, HEY1, SMARCA4, SMARCD3	0.833	0
	ATPase, HCM, PKA, TNNI3, Troponin_C, Troponin_T, cMyBP-C, calcium2+	0.607	0
	(R)-lipoic acid, CYREN, Leu-Val, NR3C2	0.833	0
	JPH2, RYR2, calcium2+	1.000	0
	Actin, CLEC3B, Troponin_C, calcium2+	1.000	0
	Ala-Pro, potassium1+, sodium1+	1.000	0
	AMPK, ATP, STK11, adenosine 5′-monophosphate, metabolic process	0.700	0
	Actin, MYBPC3, MyBP-C, PRH1, cMyBP-C, filament	0.733	0
	NFAT, PPP3, cyclosporin A	1.000	0
	Death, Leu-Val, PIMREG, atenolol	0.833	0
	Actin, MYBPC3, Myosin_complex, PRH1, PRH2, TTN, filament	0.619	0
	NFkappaB, RELA, SIRT1, SIRT6, TP53, apoptotic process	0.600	0
	Actin, RHOA, TGFB	1.000	0
	ATP2A2, CAMK2_complex, ERK, PLN, SERCA2a, calcium2+	0.600	0
	AGT, INS, apoptotic process, dioxygen, inflammatory response, nitric oxide	0.600	0
	ATP2A2, PLN, calcium2+	1.000	0
	CXCR4, SDF-1alpha, cell population proliferation	1.000	0
	CALM, CAMK2_complex, RYR2, calcium2+	1.000	0
	Ala-Pro, Death, calcium2+, isoprenaline	0.833	0
	NFAT, Phosphatase, calcium2+, sodium atom	0.833	0
	MDM2, SIRT1, TP53, apoptotic process	0.833	0
	AMPK, SLC2A1, glucose	1.000	0
	AMPK, CAB39, STK11, adenosine 5′-monophosphate, miR-195	0.800	0
	ATPase, Actin, ERVK-48, myosin heads	0.833	0
	HIF1A, glucose, lactate, metabolic process	0.833	0

	E2F1, TRIM55, TRIM63, TTN	0.833	0
	JNK, MAP3K7, USP4(muts: (C, 311, A))	1.000	0
	3′,5′-cyclic AMP, ADRB, AVP, beta2AR, diacylglycerol 30:1	0.700	0
	AKT, GSK3B, beta-AR	1.000	0
	SMAD6, TGFB, signaling	1.000	0
	Death, Leu-Val, ethanol	1.000	0
	ADP, ATP, CHKB, creatine, reactive oxygen species	0.700	0
	Actin, MyBP-C, PRH1	1.000	0
	ACK2, CSNK2A1, HDAC2, None, nucleus	0.833	0
	PLN, SERCA, calcium2+, phospholamban	0.833	0
	SMAD, SMAD7, TGFB, signaling	0.833	0
	CAMK2_complex, PKA, SERCA, calcium2+, phospholamban	0.700	0
	3′,5′-cyclic AMP, AVP, diacylglycerol 30:1	1.000	0
	AGT, CCN2, Collagen, TGFB	1.000	0
	LAMP2, MTOR, autophagy	1.000	0
	Troponin, calcium atom, calcium2+, diltiazem	0.833	0
	Death, Leu-Val, triacetylcellulose	1.000	0
	AMPK, SIRT1, resveratrol	1.000	0
	Collagen, POSTN, TGFB, cell differentiation, cell population proliferation	0.700	0
	ATPase, F_actin, Myosin_complex, tropomyosin	0.833	0
	CTNNB1, Wnt, plakoglobin	1.000	0
	CAMK2_complex, CaMKIIdelta, HDAC4, calcium2+	0.833	0
	PKA, PLN, TNNI3, TnI, Troponin, Troponin_C, cMyBP-C, calcium2+	0.607	0
	DCM, Leu-Val, doxorubicin	1.000	0
	DCM, DMD, calcium2+	1.000	0
	PRKG, cGMP, sildenafil	1.000	0
	AKT, ERK, trichostatin A	1.000	0

hypertrophic cardiomyopathy ∩ heart failure Node degree	Members	Cliqueness	# of children
	Actin, DCM, Death, PKA, Troponin, Troponin_C, cMyBP-C, calcium2+	0.607	0
*	Actin, MYBPC3, Myosin_complex, TTN, Troponin, Troponin_C, cMyBP-C, filament	0.643	2
Child of *	ATPase, Actin, MYBPC3, Myosin_complex, TTN, Troponin, Troponin_C, cMyBP-C, calcium2+, filament	0.600	0
Child of *	Actin, DCM, MYBPC3, Myosin_complex, TTN, Troponin, Troponin_C, cMyBP-C, filament	0.611	0

#	(R)-lipoic acid, CFH, Death, Leu-Val, PSMD4	0.900	2
@<br>Child of #	(R)-lipoic acid, CFH, Death, Leu-Val, PSMD4, SCD	0.800	2
Child of @	(R)-lipoic acid, CFH, DCM, Death, Leu-Val, PSMD4, SCD, cMyBP-C	0.607	0
Child of @	(R)-lipoic acid, CFH, Death, Leu-Val, PSMD4, SCD, inflammatory response	0.619	0
Child of #	(R)-lipoic acid, CFH, Death, LIAS, Leu-Val, NR3C2, PSMD4	0.619	0
	CAPN, apoptotic process, calcium2+	1.000	0
	DCM, Death, Leu-Val, Myosin_complex, Troponin_C, cMyBP-C, calcium2+	0.619	0
	AKT, AMPK, MTOR	1.000	0
	AGT, apoptotic process, dioxygen, inflammatory response, triacetylcellulose	0.700	0
	AKT, IGF1, apoptotic process	1.000	0
	(R)-lipoic acid, DCM, Death, HCM, Leu-Val, PSMD4, calcium2+	0.619	0
	EDN1, ERK, NPPA, PKC, PLN, calcium2+	0.600	0
	CAMK2_complex, CFH, CaMKIIdelta, PLN, RYR2, calcium2+, sodium atom	0.619	0
	PKA, PLN, TNNI3, TnI, Troponin, Troponin_C, cMyBP-C, calcium2+	0.607	0
	ATP, Actin, NADH, S1, calcium2+	0.700	0
	Actin, RHOA, TGFB	1.000	0
	AMPK, cell population proliferation, glucose	1.000	0
¤	Actin, MYBPC3, Myosin_complex, PKA, TTN	0.800	2
Child of ¤	Actin, DCM, MYBPC3, Myosin_complex, PKA, TTN	0.667	0
Child of ¤	Actin, MYBPC3, Myosin_complex, PKA, PRH1, TTN, filament	0.667	0
	NFkappaB, TNF, apoptotic process, p38, transcription, DNA-templated	0.700	0
	Ala-Pro, CAMK2_complex, CFH, I NaL, PKC, calcium2+, sodium atom, sodium1+	0.607	0
	ATP, INS, apoptotic process, dioxygen, glucose, reactive oxygen species	0.600	0
	ATPase, Actin, DCM, Myosin_complex, PKA, TNNI3, Troponin, Troponin_C, Troponin_T, calcium2+	0.600	0
	AMPK, ATP, INS, cell population proliferation, glucose, reactive oxygen species	0.667	0
	ERK, MYL12A, PKC, cMyBP-C, calcium2+, sodium atom	0.600	0
	NFkappaB, SIRT1, SIRT6, TNF, apoptotic process, inflammatory response	0.600	0
	AGT, AMPK, INS, apoptotic process, glucose, reactive oxygen species	0.600	0
	AGT, Angiotensin-2, Collagen, POSTN, TGFB, losartan	0.600	0

Actin, DCM, Leu-Val, PKA, cMyBP-C, calcium2+	0.600	0
NFkappaB, RELA, SIRT1, SIRT6, TP53, apoptotic process	0.600	0
Actin, MYBPC3, MYL12A, Myosin_complex, PRH1, Troponin, cMyBP-C, calcium2+, filament	0.639	0
LAMP2, MTOR, autophagy	1.000	0
CAMK2_complex, CFH, DCM, I NaL, Leu-Val, RYR2, calcium2+, sodium atom	0.607	0
BMP10, SMARCA4, cell population proliferation	1.000	0
CALM, CAMK2_complex, CFH, DCM, JPH2, RYR2, calcium2+	0.667	0
3′,5′-cyclic AMP, Actin, Myosin_complex, PKA, Troponin, Troponin_C, calcium2+, filament	0.607	0
EDN1, GATA4, NFAT, Phosphatase, calcium2+	0.700	0
ATPase, MYL12A, Myosin_complex, PKC, calcium2+, filament	0.667	0
ATPase, Actin, F_actin, MYL12A, Myosin_complex, TNNI3, Troponin_C, calcium2+	0.607	0
DCM, PRH1, TNNI3, TnI, Troponin_C, Troponin_T, calcium2+	0.619	0
ATP2A2, CAMK2_complex, ERK, PKA, PLN, calcium2+	0.600	0
Angiotensin-2, Collagen, TGFB, cell population proliferation	0.833	0
Death, Leu-Val, triacetylcellulose	1.000	0
ADRB, GRK2, Gly-Gln, PKC, calcium2+	0.700	0
Ala-Pro, Death, calcium2+, isoprenaline	0.833	0
ATPase, HCM, PKA, TNNI3, Troponin_C, Troponin_T, cMyBP-C, calcium2+	0.607	0
Death, Leu-Val, PIMREG, atenolol	0.833	0
Actin, PKA, PKC, TnI, Troponin_C, Troponin_T, calcium2+	0.667	0
HIF1A, glucose, lactate, metabolic process	0.833	0
Actin, MYBPC3, MyBP-C, PRH1, cMyBP-C, filament	0.733	0
DCM, Death, HCM, MYH7, TNNI3, calcium2+	0.667	0
AKT, ERK, trichostatin A	1.000	0
Ala-Pro, Troponin_T, calcium2+, isoprenaline, sodium atom, sodium1+	0.667	0
Actin, F_actin, Myosin_complex, Troponin, Troponin_T, calcium2+, tropomyosin	0.619	0
SMAD, SMAD6, SMAD7, TGFB, signaling	0.700	0
AGT, INS, glucose, nitric oxide	0.833	0
Ala-Pro, Ranolazine, calcium2+, potassium1+, sodium atom, sodium1+	0.667	0
(R)-lipoic acid, CYREN, Leu-Val, NR3C2	0.833	0
MDM2, SIRT1, TP53, apoptotic process	0.833	0
DPF3a, HEY1, SMARCA4, SMARCD3	0.833	0
Death, Leu-Val, ethanol	1.000	0

	Death, Leu-Val, PSMD4, SCD	1.000	0
	CAMK2_complex, CAMKIIdelta, HDAC4, calcium2+	0.833	0
	ATPase, F_actin, Myosin_complex, tropomyosin	0.833	0
	AMPK, ATP, STK11, adenosine 5′-monophosphate, metabolic process	0.700	0
	3′,5′-cyclic AMP, ADRB, AVP, beta2AR, diacylglycerol 30:1	0.700	0
	EDN1, ERK, NFAT, NPPA, calcium2+	0.700	0
	ERK, MEK, RAF, RAF1, microtubule	0.700	0
	SMAD, SMAD7, TGFB	1.000	0
	E2F1, TRIM55, TRIM63, TTN	0.833	0
	PRKAG2, adenosine 5′-monophosphate, glycogen	1.000	0
	DCM, DMD, calcium2+	1.000	0
	CALM, CAMK2_complex, RYR2, calcium2+	1.000	0
	TLX2, calcium2+, sodium atom	1.000	0
	NFAT, PPP3, cyclosporin A	1.000	0
	AMPK, CAB39, STK11, adenosine 5′-monophosphate, miR-195	0.800	0
	Collagen, POSTN, TGFB, cell differentiation, cell population proliferation	0.700	0
	AKT, GSK3B, beta-AR	1.000	0
	NFAT, Phosphatase, calcium2+, sodium atom	0.833	0
	CAMK2_complex, PKA, SERCA, calcium2+, phospholamban	0.700	0
	(R)-lipoic acid, LIAS, Leu-Val	1.000	0
	ACK2, CSNK2A1, HDAC2, None, nucleus	0.833	0
	CXCR4, SDF-1alpha, cell population proliferation	1.000	0
	ATP2A2, PLN, calcium2+	1.000	0
	ADP, ATP, CHKB, creatine, reactive oxygen species	0.700	0
	BIN1, CAV1, calcium2+	1.000	0
	AMPK, SIRT1, resveratrol	1.000	0
	JPH2, RYR2, calcium2+	1.000	0
	AGT, CCN2, Collagen, TGFB	1.000	0
	Actin, MyBP-C, PRH1	1.000	0
	Actin, CLEC3B, Troponin_C, calcium2+	1.000	0
	Troponin, calcium atom, calcium2+, diltiazem	0.833	0
	ATPase, Actin, ERVK-18, myosin heads	0.833	0
	PRKG, cGMP, sildenafil	1.000	0
	CTNNB1, Wnt, plakoglobin	1.000	0
	DCM, Leu-Val, doxorubicin	1.000	0
	Ala-Pro, potassium1+, sodium1+	1.000	0
	JNK, MAP3K7, USP4(muts: (C, 311, A))	1.000	0
	AMPK, SLC2A1, glucose	1.000	0
	PLN, SERCA, calcium2+, phospholamban	0.833	0

hypertrophic cardiomyopathy ∩ major adverse cardiovascular events Page rank	Members	Cliqueness	# of children
	(R)-lipoic acid, Leu-Val, NR3C2	1.000	0

hypertrophic cardiomyopathy ∩ major adverse cardiovascular events Node degree	Members	Cliqueness	# of children
	(R)-lipoic acid, Leu-Val, NR3C2	1.000	0

hypertrophic cardiomyopathy ∩ rehospitalization, page rank – none

hypertrophic cardiomyopathy ∩ rehospitalization, node degree – none
