# Supplementary material for: Shared Molecular Mechanisms of Hypertrophic Cardiomyopathy and Its Clinical Presentations: Automated Molecular Mechanisms Extraction Approach
Source: Life (Basel). 2021 Aug 3;11(8):785. doi: 10.3390/life11080785 (PMC8398249; doi:10.3390/life11080785)
Supplement: Supplementary file 1 [file life-11-00785-s001.zip › Supplementary Table S1.pdf]

Table S1. The most important nodes in the networks. Elements are stated in descending order of importance. K-shell decomposition is a method that ranks the most important nodes in a network and partitions them into shells based on that rank. The elements are presented in the form that they were extracted in.

Network	95th Percentile Shell	90th Percentile Shell	85th Percentile Shell	80th Percentile Shell
hypertrophic cardiomyopathy, cardiomyocyte hypertrophy	calcium2+, apoptotic process, AGT, AKT, AMPK, None→nucleus†, ERK, Collagen	cell population proliferation, TGFB, SIRT1, CAMK2_complex, PLN, PKA, NFAT, EDN1	bisphenol A, Phosphatase, Death, ATP2A2, CTNNB1, ATPase, ATP, caffeine	2-(3,4-dimethoxyphenyl)-5-\\{[2-(3,4-dimethoxyphenyl)ethyl](methyl)amino\\}-2-(propan-2-yl)pentanenitrile, CLEC3B, RYR2, CALM, ADRB, sarcoplasmic reticulum→None†, TLX2, DNA-templated transcription
hypertrophic cardiomyopathy, myofibrillar disarray	Actin, MAPK7, RAF1S257L, MYL12A, Myosin_complex , MYBPC3, EDN1, ATP, RAF1, PD98059, cyclosporin A, ERK	MyHC 723, filament, MEF2, PPP3, NFAT, TM, MAPK3, Troponin_C, Myosin_complex(bound: [Actin, True], calcium2+, RAF1S257L iCMs, ATPase	Hypertrophy, ATPase activity, NAT9, app / (f app + g app, alpha-actinin, Mg-ATPase, hydrolysis, MAP2K5, Disease, mutant iCMs, ERK5 auto-phosphorylation, MEK, adenosine 5'-monophosphate	Translocation, activation pathway, AMPK, DUSP6, Ad, phosphorylation TEY site kinase domain ERK5 known be kinase, RAF1 translocate cardiomyocyte nucleus, HCM iPSC, PKA, AKT mTOR p38-MAPK JNK-MAPK signaling, contractility, PRH1
hypertrophic cardiomyopathy, cardiomyocyte disarray	-	Myosin_complex	-	ATPase
hypertrophic cardiomyopathy, myocardial remodeling	calcium2+, Collagen, apoptotic process, AGT, TGFB	cell population proliferation, CAMK2_complex, TNF, inflammatory response, Angiotensin-2	sodium atom, Death, ATP, CALM, MYL12A	AKT, SNCG, SH2B2, ATPase, caffeine, RYR2
hypertrophic cardiomyopathy, cardiac remodeling	calcium2+, apoptotic process, AMPK, AGT, AKT, TGFB, cell population proliferation, Collagen, Actin, reactive oxygen species, SIRT1	Glucose, INS, None→nucleus†, inflammatory response, ERK, NFkappaB, p38, TNF, DNA-templated transcription, CAMK2_complex, PLN	PKA, sodium atom, RYR2, ATPase, RyR, SERCA, CALM, Death, NFAT, Troponin_C, metabolic process	SNCG, nifedipine, homeostatic process, sodium1+, Troponin, phospholamban, procaine, bradykinin, sarcoplasmic reticulum→None†, caffeine, Phosphatase, Hydrogen-Ion Concentration
hypertrophic cardiomyopathy, myocardial fibrosis	calcium2+, apoptotic process, AGT, TGFB, Collagen, Leu-Val*, cell population prolifera-	CCN2, p38, DCM, AKT, NFkappaB, losartan, TNF, Neoplasm Invasiveness, TGFB1, SMAD	Glucose, DNA-templated transcription, reactive oxygen species, SIRT1,	Dioxygen, GOPC, CYCS, ATP2A2, EGFR, FSD1, TP53, ATP, RyR, SH2B2

	tion, inflammatory response, AMPK, Angiotensin-2		CAMK2_complex, sodium atom, Death, SMAD7, IGF1, TLX2	
hypertrophic cardiomyopathy, left ventricular outflow tract obstruction	Leu-Val*, calcium2+, Left ventricular outflow tract obstruction LVOTO systolic anterior motion SAM anterior mitral leaflet	TGFB, POSTN, 2-(3,4-dimethoxyphenyl)-5-\\{[2-(3,4-dimethoxyphenyl)ethyl](methyl)amino\\}-2-(propan-2-yl)pentanenitrile	Collagen, Death, PIMREG, SNCG	V1-3, dobutamine, MYK-461
hypertrophic cardiomyopathy, impaired myocardial relaxation	-	-	-	TGFB
hypertrophic cardiomyopathy, myocardial stiffness	TTN, Actin	TGFB, PRKCA	PRKG, RING1	RBM20, TRIM63
hypertrophic cardiomyopathy, diastolic dysfunction	calcium2+, Actin, Leu-Val*, TTN, cMyBP-C, apoptotic process, filament, PKA, DCM, sodium atom, CAMK2_complex, TGFB, AMPK, PLN, TnI	Myosin_complex, Troponin_C, tropomyosin, AGT, ERVK-18, reactive oxygen species, Collagen, Troponin, ATP, MyBP-C, Ala-Pro*, RYR2, (-)-epigallocatechin 3-gallate, ATPase, Death	Serca2a, SNCG, (-)-epicatechin-3-O-gallate, (+)-catechin, TN-I, TLX2, sodium1+, RyR, ERK, SERCA, ATP2A2, MICU2, GATA4, Phosphatase, SERCA2a, Hydrogen-Ion Concentration	TNNT2(muts: (None, None, None)), procaine, arrhythmias, GOPC, S1, sarcoplasmic reticulum→None†, MCU, caffeine, SH2B2, sarcolemma→None†, transport, metabolic process, 2-(3,4-dimethoxyphenyl)-5-\\{[2-(3,4-dimethoxyphenyl)ethyl](methyl)amino\\}-2-(propan-2-yl)pentanenitrile, diltiazem, ruthenium red
hypertrophic cardiomyopathy, atrial fibrillation	calcium2+, PSMD4, CAMK2_complex, Leu-Val*, inflammatory response, sodium atom, AGT, Death, PLN, Ala-Pro*, RYR2, apoptotic process, JPH2, TLX2, CFH	PKA, ATP, 2-(3,4-dimethoxyphenyl)-5-\\{[2-(3,4-dimethoxyphenyl)ethyl](methyl)amino\\}-2-(propan-2-yl)pentanenitrile, caffeine, Phosphatase, ATPase, sodium1+, RyR, Troponin, phospholamban, SERCA, isoprenaline, CALM, NFAT, ERK, EDN1	ypkA, SNCG, nifedipine, CAT, PYR3, KN-93, GOPC, dobutamine, dantrolene, arrhythmias, ryanodine, (-)-epigallocatechin 3-gallate, CPVT, diltiazem, CAPN	sarcoplasmic reticulum→None†, CAV1, Hydrogen-Ion Concentration, ryanodine receptor, N-[2-(4-bromocinnamylamino)ethyl]isoquinoline-5-sulfonamide, isocyanic acid, SERCA2a, CHF, CaMKIIdelta, Blebbistatin, CYBB, RyRs, PPP2, pyraclofos, FSD1, TGFB
hypertrophic cardiomyopathy, sudden cardiac death	calcium2+, Death, Actin, DCM, SCD, Leu-Val*, RYR2, sodium atom, CAMK2_complex, HCM, GSTK1, Myosin_complex, Troponin, ATP, PKA, TNNT1(muts: (R, 97,	ATP2A2, filament, tropomyosin, sodium1+, Serca2a, SNCG, nifedipine, homeostatic process, 2-(3,4-dimethoxyphenyl)-5-\\{[2-(3,4-dimethoxy-	N-[2-(4-bromocinnamylamino)ethyl]isoquinoline-5-sulfonamide, TNNT1(muts: (F, 88, L)), isocyanic acid, ryanodine, EDN1, CAPN, PPP2, pyraclofos, PSMD4, MYH7,	TTN, malignant ventricular arrhythmia, ICD implants, alpha-MHC, amiodarone, ARVC, ER, death related congestive heart cardiovascular disease,

	L)), CFH, Troponin_C, PLN, Ala-Pro*, Troponin_T, MYL12A, isoprenaline, GJA1, TNNI3	phenyl)ethyl](methyl)amino\\}-2-(propan-2-yl)pentanenitrile, HCM Phenotype, caffeine, KN-93, RyR, phospholamban, ADRB, SERCA, CALM, enhancer factor2, MYLK, dantrolene, sarcoplasmic reticulum→None†, N-carbamoylsarcosine, MYBPC3(muts: (None, None, None)), m., calsequestrin, TLX2	miR-139-5p, cell population proliferation, cMyBP-C, dioxygen, TGFB, LGE, PRH1, Collagen, ARSA, AKT, Death Sudden Cardiac, ACOD1, beta-MHC, (R)-lipoic acid, mlc-4, CM	(-)-epicatechin-3-O-gallate, TNNT1, TM, ADP, S1, GH1, metabolic process, ATPase, RHOA, ERVK-18, ATPase activity, myosin heads, CTN, KRT3, XIRP1, alpha-actinin, reactive oxygen species, Myosin
hypertrophic cardiomyopathy, coronary microvascular dysfunction	-	calcium2+	DCM	pyraclofos
hypertrophic cardiomyopathy, myocardial ischemia	calcium2+, apoptotic process, AMPK, reactive oxygen species, AKT, ATP, glucose, SIRT1, Leu-Val*, NFkappaB, ERK, inflammatory response, INS, PKA, TNF	Death, metabolic process, filament, PLN, sodium atom, RYR2, CAMK2_complex, Ranolazine, sodium1+, cMyBP-C, RyR, Troponin, CAPN, Troponin_C, ATP2A2	SNCG, (-)-epicatechin-3-O-gallate, nifedipine, KN-93, NOS1, phospholamban, SERCA, CALM, NFAT, GJA1, calcium atom, sarcoplasmic reticulum→None†, Wnt, EDN1, TLX2, caffeine	MCU, Phosphatase, CAV1, Hydrogen-Ion Concentration, (-)-epigallocatechin 3-gallate, LTCC, ogt-1, ATPase, 2-(3,4-dimethoxyphenyl)-5-\\{[2-(3,4-dimethoxyphenyl)ethyl](methyl)amino\\}-2-(propan-2-yl)pentanenitrile, CLEC3B, diltiazem, Protease, PPP2, pyraclofos, FSD1
hypertrophic cardiomyopathy, heart failure	calcium2+, Actin, apoptotic process, Leu-Val*, DCM, AMPK, AGT, Death, ERK, AKT, PKA, Myosin_complex, TTN, CAMK2_complex, DNA-templated transcription, TGFB, cell population proliferation, sodium atom, ATP, reactive oxygen species, Troponin_C, cMyBP-C, PKC, glucose, filament, None→nucleus†, SIRT1, NFkappaB, INS, Collagen, inflammatory response, CFH, Tro-	Dioxygen, homeostatic process, CHF, RyR, tricarboxylic acid cycle, S100A9, calcium atom, Hydrogen-Ion Concentration, JPH2, NOS3, ryanodine, (-)-epigallocatechin 3-gallate, sarcoplasmic reticulum→None†, SNCG, REM1, ATP2A2, cCTnC, SERCA2a, CAV1, CPVT, BIN1, EMD, sarcolemma→None†, NADH, I Ca-L, TLX2, 2-(3,4-dimethoxyphenyl)-5-\\{[2-(3,4-di-	Triacetylcellulose, BCL2, Angiotensin-2, cell differentiation, (R)-lipoic acid, SIRT6, SCD, PIMREG, HDAC, losartan, nitric oxide, ethanol, TNF, HIF1A, I NaL, phenylephrine, CTNNB1, TP53, activity, TGFB1, atenolol, trichostatin A, NR3C2, NPPA, F_actin, CSRP3, adenosine 5'-monophosphate, PPARA, diacylglycerol 30:1, POSTN, FBXO32, signaling, SMAD, RHOA, necrotic cell death, cel-	LIAS, HIF, aldosterone, myosin heads, Ischemia, ERBB2, FOXO1, myosin, TN, cell death, EPAS1, TNT, XIRP1, TM, triglyceride, PFN, release, C0C1f, assays, Cofilin, triphosphatase activity, Myosin, hydrolysis, UTRN, MEK, microtubule, RAF, ADP, BAG3, MDM2, beta-AR, lactate, TG, PRPF6, extracellular matrix, JNK, CARL, SULT1E1, FERMT2, miR-133, gp130 receptor, GSK3B, etoposide, PPIF, CYREN, RTN4,

	ponin, RYR2, 3',5'-cyclic AMP, ATPase, Troponin_T, MYL12A, autophagy, NFAT, SMARCA4, TNNI3, TnI, metabolic process, PSMD4, PLN, HCM, tropomyosin, sodium1+, E2F1, Phosphatase, MYBPC3, MTOR, isoprenaline, Ala-Pro*, ADRB, S1, p38, CaMKIIdelta, CLEC3B, transport, DMD, GATA4, phospholamban, ISO, EDN1, CALM	methoxyphenyl)ethyl](methyl)amino\\}-2-(propan-2-yl)pentanenitrile, Ser-Leu*, AIP-II, diltiazem, RyRs, Protease, CAPN, PPP2, nifedipine, EC, Dobutamine, SERCA, Proteasome, dantrolene, CCL5, ryanodine receptor, LTCC, ssTnI, CYBB, calsequestrin, ruthenium red, pyraclofos, FSD1, KN-93, NOS1, phosphoprotein, GOPC, terms ARVC mechanisms DSG induce electrical coupling cardiac myocytes lead myocyte cell death Azaouagh Erbel Pilichou et al ., RYR2, SH2B2, CXCL12, N-[2-(4-bromocinnamylamino)ethyl]isoquinoline-5-sulfonamide, cNTnC, EF-hand, Ranolazine, phenazine-1-carboxylate, EEF2, caffeine, N-carbamoylsarcosine, CAMK, PRH1, GJA1, MYC	lular component biogenesis, PPARGC1A, AVP, Cell Survival, CCN2, MyBP-C, SLC4A3, Mhc, IGF1, PRKG, HEY1, resveratrol, doxorubicin, FGF23, proteolysis, mdfA, cGMP, TXNDC5, TRIM63, TRIM55, miR-195, STK11, CAB39, ANPEP, mTORC1, beta-MHC, Neoplasm Invasiveness, FOXO3, ERVK-18, nppb, SLC2A1, SMAD7, ATPase activity	SLC25A6, HMBS, MAP3K1, mtPTP, APEX1, CYP2E1, proteasome inhibitors, Arg-Val*, crt, LAD1, RENBP, 17alpha-ethynylestradiol, nedocromil, FASLG, CYCS, N(gamma)-nitro-L-arginine methyl ester, polonium atom, Caspase, PRKAG2, Gly-Gln*, SMARCD3, endoplasmic reticulum
hypertrophic cardiomyopathy, major adverse cardiovascular events	calcium2+	Leu-Val*, (R)-lipoic acid	NR3C2, Arg-Val*	DNA-templated transcription, ATPase
hypertrophic cardiomyopathy, rehospitalization	-	Death	-	PSMD4

* Abbreviations in HCM literature that are falsely turned into amino acid sequences by reading systems (Leu-Val = LV, commonly used for left ventricle; Ala-Pro = AP, used for action potential; Ser-Leu = SL, abreviation for semilunar (valves); Arg-Val = RV, used for right ventricle; Gly-Gln = GQ)

† transport (from→to)
